# Probing the local structure of Bi_2_O_3_ chemical derivatives: the neglected cation sublattice

**DOI:** 10.1107/S2052520625009400

**Published:** 2025-11-24

**Authors:** Sikhumbuzo M. Masina, Gugulethu C. Nkala, Kevin H. Stone, Daniel Olds, Caren Billing, David G. Billing

**Affiliations:** a Molecular Science Institute, School of Chemistry University of the Witwatersrand Private Bag X3 Johannesburg 2050 South Africa; b DSI-NRF Centre of Excellence in Strong Materials University of the Witwatersrand Private Bag X3 Johannesburg 2050 South Africa; c Stanford Synchrotron Radiation Lightsource SLAC National Accelerator Laboratory 2575 Sand Hill Road Menlo Park CA94025 USA; d National Synchrotron Light Source II Brookhaven National Laboratory Upton NY11973 USA; University of Geneva, Switzerland

**Keywords:** local structure, total scattering data, X-ray absorption spectroscopy, δ phase

## Abstract

High-resolution synchrotron X-ray diffraction data confirm the stabilization of the average structure at ambient temperatures as a defect fluorite structure in ternary and quaternary oxides of Bi_2_O_3_, where some Bi cations are replaced with Dy^3+^ and/or Er^3+^, Nb^5+^ and W^6+^. Rietveld refinement of the diffraction data indicates that when the larger cation Er^3+^ (0.89 Å) is replaced with smaller cations W^6+^ (0.42 Å) and/or Nb^5+^ (0.48 Å), the unit-cell parameter *a*, based on the relative ionic radii of the substituents, counterintuitively increases. Total scattering data show that the identity of the cation substituent is important in determining the positions of some Bi cations in the defect fluorite structure. Er^3+^ and Dy^3+^ cations induce monoclinic-like distortions that extend only locally in the Bi^3+^ sublattice. X-ray absorption spectroscopy provides evidence that some Dy cations, in the δ phases, prefer to keep a similar local environment to that in the parent Dy_2_O_3_. W cations are found to be predominantly tetrahedrally coordinated in the δ phases explored in this work.

## Abstract 2

The importance of the cation sublattice in influencing both the average and local structures of δ-Bi_2_O_3_-like phases is highlighted. It also reveals the inadequacy of conventional average structural analysis methods in describing comprehensively the structure of real materials.

## 1. Introduction

In-depth knowledge of both the average and local structural details of functional materials is a prerequisite to establishing reliable correlations with physical properties and paves the way to designing advanced materials with enhanced properties. One of the functional materials that has found extensive use in the fabrication of devices of technological importance is Bi_2_O_3_ (Cabot *et al.*, 2004 ▸; Bhande *et al.*, 2011 ▸; Li *et al.*, 2014 ▸; Won-In *et al.*, 2011 ▸). Bi_2_O_3_ exists in many different polymorphs. One of the four main polymorphs is the monoclinic α-Bi_2_O_3_ phase with the *P*2_1_/*c* space-group type, which is the thermodynamically stable polymorph from ambient temperature to 729°C (Harwig & Gerards, 1978 ▸). Beyond this temperature, the α-Bi_2_O_3_ phase transitions into the cubic defect fluorite δ-Bi_2_O_3_ phase (*Fm*3*m*) and Bi_2_O_3_ melts at 825°C (Harwig & Gerards, 1978 ▸). Depending on the cooling conditions, the δ-Bi_2_O_3_ phase can either transition into the tetragonal β-Bi_2_O_3_ phase (*P*42_1_*c*) at ∼650°C or into the body centred cubic γ-Bi_2_O_3_ phase (*I*23) at ∼640°C before reverting to the α-Bi_2_O_3_ phase (Sammes *et al.*, 1999 ▸; Levin & Roth, 1964 ▸). The β-Bi_2_O_3_ structure has been reported to have essentially the same cation sublattice as the δ-Bi_2_O_3_ phase with the oxygen vacancies being ordered along the equivalent of the 〈100〉 direction in the fluorite phase (Blower & Greaves, 1988 ▸).

In its δ-Bi_2_O_3_ phase, Bi_2_O_3_ has the highest ionic conductivity (≥ 1 S cm^−1^) ever reported for an oxide ion conductor in its narrow stable temperature range (730–824°C) (Harwig & Gerards, 1978 ▸). To stabilize δ-Bi_2_O_3_-like phases at ambient temperature, henceforth referred to as stabilized δ phases, isovalent and aliovalent cations have been used extensively to substitute the Bi^3+^ cations albeit with a moderate decrease in ionic conductivity (Jiang *et al.*, 2002 ▸; Watanabe & Sekita, 2005 ▸; Mercurio *et al.*, 1990 ▸; Portefaix *et al.*, 1997 ▸; Shitara *et al.*, 2017 ▸; Bandyopadhyay & Dutta, 2018 ▸). The extent to which the conductivity is decreased depends on the identity of the cation and the total substituent concentration. Factors such as decrease in both polarizability and ionic radius of the substituent, trapping of vacancies and vacancy–substituent clustering have been reported to contribute to the decrease in conductivity observed in solid solutions (Kharton *et al.*, 2004 ▸; Borowska-Centkowska *et al.*, 2018 ▸; Laarlf & Theobald, 1986 ▸; Boyapati *et al.*, 2001 ▸). The average structures of the stabilized δ phases are also described by the *Fm*3*m* space-group type, where the metal cations share the 4*a* site (0 0 0), while the oxygen ions are distributed among three different crystallographic sites, namely, the 8*c* site (0.25 0.25 0.25), the 32*f* site (*x* *x* *x*), where *x* ∼ 0.3 and the 48*i* site (0.5 *y* *y*) where *y* ∼ 0.2 (Abrahams *et al.*, 2010 ▸). The 48*i* site is an interstitial site and there is no evidence of its occupation in the parent δ-Bi_2_O_3_ phase (Hull *et al.*, 2009 ▸; Battle *et al.*, 1983 ▸; Yashima & Ishimura, 2003 ▸), its occupation is attributed to the substituents.

The stabilized δ phases obtained by substituting Bi^3+^ with lanthanides such as Dy^3+^ and Er^3+^ have been shown to be highly conductive but metastable and exhibit phase separation upon prolonged annealing at temperatures ≤600°C (Verkerk *et al.*, 1980 ▸; Verkerk & Burggraaf, 1981 ▸; Jung *et al.*, 2010 ▸). The oxide ions and oxygen vacancies order in these systems and ionic conductivity decreases significantly as a result. These problems are normally circumvented by adding low concentrations of supervalent cations such as W^6+^, Nb^5+^ and Hf^4+^ (Shitara *et al.*, 2017 ▸; Yun *et al.*, 2017 ▸), a strategy adopted in this work. Systems fabricated in this way exhibit both high conductivity and stability relative to the singly substituted systems. In this work, the systems (Bi_2_O_3_)_1–*x*–*y*_(Dy_2_O_3_)_*x*_(Er_2_O_3_)_*y*_, (Bi_2_O_3_)_1–*x*–*y*–*z*_(Dy_2_O_3_)_*x*_(Er_2_O_3_)_*y*_(WO_3_)_*z*_,(Bi_2_O_3_)_1–*x*–*y*–*z*_(Dy_2_O_3_)_*x*_(Er_2_O_3_)_*y*_(Nb_2_O_5_)_*z*_ and (Bi_2_O_3_)_1–*x*–*y*–2*z*_(Dy_2_O_3_)_*x*_(Nb_2_O_5_)_*y*_(WO_3_)_2*z*_ were fabricated to stabilize the δ phase and study the effects of the substituents in the cation sublattice which have been neglected in the literature (Shitara *et al.*, 2017 ▸; Verkerk *et al.*, 1980 ▸; Verkerk & Burggraaf, 1981 ▸; Jung *et al.*, 2010 ▸; Yun *et al.*, 2017 ▸).

The local structure of the stabilized δ phases is a subject of intense research, with the focus on the distribution of the oxide ions around the metal cations, *i.e.* the metal–oxygen (*M*–O) correlations (Abrahams *et al.*, 2010 ▸; Hull *et al.*, 2009 ▸; Borowska-Centkowska *et al.*, 2020 ▸; Liu *et al.*, 2011 ▸). Very few studies have been done on the metal–metal (*M*–*M*) correlations (Leszczynska *et al.*, 2013 ▸; Borowska-Centkowska *et al.*, 2019 ▸). Therefore, this work seeks to bridge this gap and provide a complementary picture of the local structure of the stabilized δ phases. To this end, total scattering data, the X-ray pair distribution function (PDF) technique and X-ray absorption spectroscopy have been used to probe the metal–metal correlations and the local environment around a specific substituent to gain insight into the local structure of the material around the metal cations.

## 2. Experimental methods

### 2.1. Synthetic methods

Samples of (Dy_2_O_3_)_*x*_(Er_2_O_3_)_*y*_(Bi_2_O_3_)_1–*x*–*y*_ (here called 100xD100yESB), (Dy_2_O_3_)_*x*_(Er_2_O_3_)_*y*_(WO_3_)_*z*_(Bi_2_O_3_)_1–*x*–*y*–*z*_ (100xD100yE100zWSB), (Dy_2_O_3_)_*x*_(Er_2_O_3_)_*y*_(Nb_2_O_5_)_*z*_(Bi_2_O_3_)_1–*x*–*y*–*z*_ (100xD100yE100zNSB) and (Dy_2_O_3_)_*x*_(Nb_2_O_5_)_*y*_(WO_3_)_2*z*_(Bi_2_O_3_)_1–*x*–*y*–2*z*_ (100xD100yN200zWSB), where 0.08 ≤ *x* ≤ 0.16, 0.04 ≤ *y* ≤ 0.08 and 0 ≤ *z* ≤ 0.02 were prepared via the solid state reaction method. Bi_2_O_3_ (Sigma-Aldrich, nano, 99.999% pure), Er_2_O_3_ (Sigma-Aldrich, 99.999% pure), and WO_3_ (Acros Organics, 99+% pure) were pre-fired at 700°C to remove any possible bis­mutite (Bi_2_O_3_·CO_2_) and moisture. These have been shown to alter the phase evolution in the formation of Bi_2_O_3_ solid solutions (Levin & Roth, 1964 ▸). Dy_2_O_3_ and Nb_2_O_5_ were obtained by thermal decomposition of Dy(NO_3_)_3_·6H_2_O (Sigma-Aldrich, 99.99% pure) and ammonium niobate(V) oxalate hydrate (Sigma-Aldrich, 99.99% pure) using the method employed by Melnikov *et al.* (2015 ▸). Stoichiometric amounts of Bi_2_O_3_, Dy_2_O_3_, Er_2_O_3_, Nb_2_O_5_ and WO_3_ were mixed in an agate mortar and manually ground for an hour in acetone (99.56% pure, KEM*i*CAL) to create the desired systems. The ground powders were dried in an oven at 80°C for 12 h before being fired in a furnace from ambient temperature to 800°C at a heating rate of ∼6°C min^−1^. The powders were then soaked at 800°C in air for 24 h before being left in the furnace to cool to ambient temperature (over a period of ∼12 h). An intermediate grinding step was performed before the powders were pressed into pellets using a uniaxial cold press at a constant pressure of 75 MPa and a 13 mm die. The pellets were then sintered at 850°C for a further 24 h before being quenched in air to ambient temperatures.

### 2.2. Diffraction data

Beamline 2-1 at the Stanford Synchrotron Radiation Light Source (SSRL) was used to collect the diffraction data (Stone *et al.*, 2023 ▸). The source is a 1.3 T bending magnet, and the diffractometer is equipped with a Huber two-circle goniometer. The radiation was monochromated using an Si (111) double-crystal monochromator. The wavelength was 0.72929 Å and the measurements were performed in transmission mode using a Kapton capillary (∼0.7 mm outside diameter). The wavelength used was chosen so that all the absorption edges of the elements (Bi, Dy, Er, Nb and W) in the samples were avoided to increase data quality. The diffractometer employs a Pilatus 100 K small area detector that was mounted at ∼700 mm distance downfield of the sample. A diamond powder (∼75–85 wt%) was mixed with the samples to act as an internal standard to account for sample displacement and reduce absorption. A μ*R* of ∼73 was reduced to μ*R* of ∼11–18 at 0.72929 Å after dilution. μ is the linear absorption coefficient of the powder and *R* the approximate capillary diameter. 2-D images were calibrated and integrated into a typical powder diffraction pattern. Sample displacement was corrected for using the method developed by Gozzo *et al.* (2010 ▸).

### 2.3. Total scattering and the PDF technique

Data for total scattering were collected on beamline 28-ID-1 at NSLS II (Brookhaven National Laboratory). The X-ray source is a damping wiggler, and the beam is focused horizontally and vertically by a side-bounce monochromator and a vertically focusing mirror, respectively. A monochromatic wavelength of 0.1671 Å in Debye–Scherrer geometry was used. The diffractometer employs a PerkinElmer area detector (200 µm × 200 µm pixel size) which was mounted at a direct distance of ∼241 mm for measuring total scattering data. Kapton capillaries with an outside diameter of 0.9 mm were used to hold the sample. LaB_6_ (NIST SRM 660c) and Si (NIST SRM 640d) were used as external calibrants for the measurements. Total scattering data were reduced using *xPDFsuite* (Yang *et al.*, 2014 ▸). The reduced PDFs were obtained by the sine Fourier transform of the *Q* weighted and reduced total scattering structure function *S*(*Q*), *i.e.*
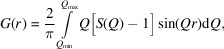
where 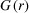 is the reduced PDF, *Q* = 4πsinθ/λ is the magnitude of the scattering vector and *r* is the radial distance. *Q*_min_ and *Q*_max_ values were 0.1 and 25.1 Å^−1^, respectively. The reduced PDFs were analysed using small box modelling as implemented in *TOPAS Academic* (Coelho *et al.*, 2015 ▸; Coelho, 2018 ▸).

### 2.4. X-ray absorption spectroscopy

BL2-2 at the SSRL was used to perform the X-ray absorption spectroscopy (XAS) measurements. The source is a bending magnet and can perform measurements in the energy range 4.5–37 keV. The energy of the unfocused radiation was tuned using a double-crystal Si (220) monochromator to scan across the *L*_3_ edges of Dy, Er and W. The measurements were taken in fluorescence mode and the samples were pelletized. Spectra were processed using the *Demeter* (Ravel & Newville, 2005 ▸) package to obtain the first- and second-derivative spectra. Only the X-ray absorption near-edge structure (XANES) part of the spectra were considered in this work.

## 3. Results and discussion

### 3.1. Average structure analysis

**Figure 1:**
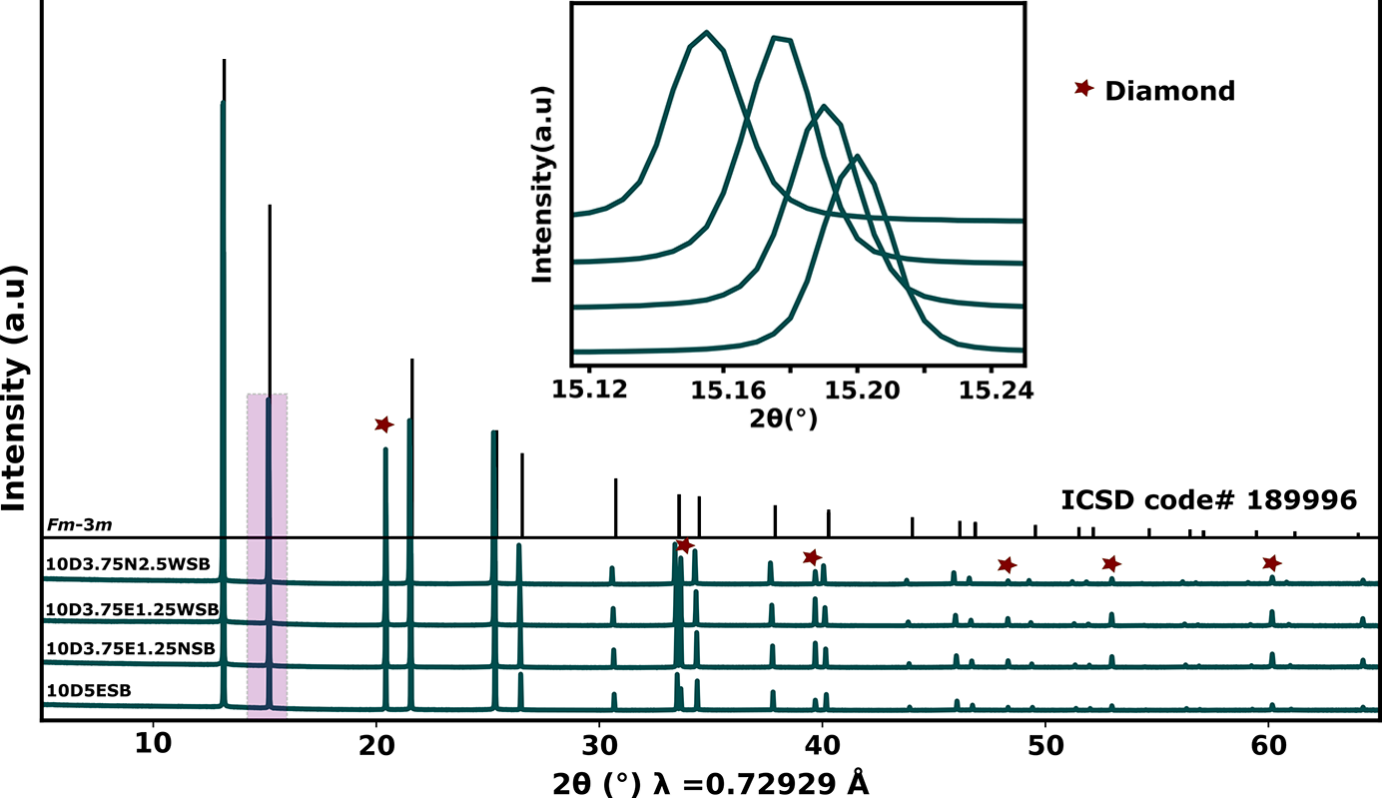
Bragg data showing that the average structure of the solid solutions belongs to the same space-group type (*Fm*3*m*) as that of δ-Bi_2_O_3_. The inset highlights significant variations in the peak positions of the second peak, but all the peaks were shifted. Diamond powder was used as an internal standard.

In all four of the synthesized systems, the δ phase was stabilized to ambient temperature (Fig. 1 ▸). No extra peaks were observed, suggesting that the materials consisted of a single crystalline phase.

Interestingly, a peak shift to lower 2θ for all the systems, where Er^3+^ was substituted partially or totally with W^6+^ and/or Nb^5+^ (Fig. 1 ▸ inset), was noted. This implied an expansion occurred in the unit cell, which is counterintuitive because the larger Er^3+^ (0.89 Å) cations were being replaced by smaller cations, W^6+^ (0.60 Å) and Nb^5+^ (0.64 Å), so a reduction in the unit cell would be expected. The ionic radii given in brackets are for the octahedrally coordinated metal ions to oxygen (Shannon, 1976 ▸), as would be expected in the cubic δ phase.

The use of the diamond internal standard allowed the direct inference from the diffraction data of the change in the unit cell with changing identity of the substituent cation by revealing the effects of capillary displacement on the peak positions.

**Figure 2:**
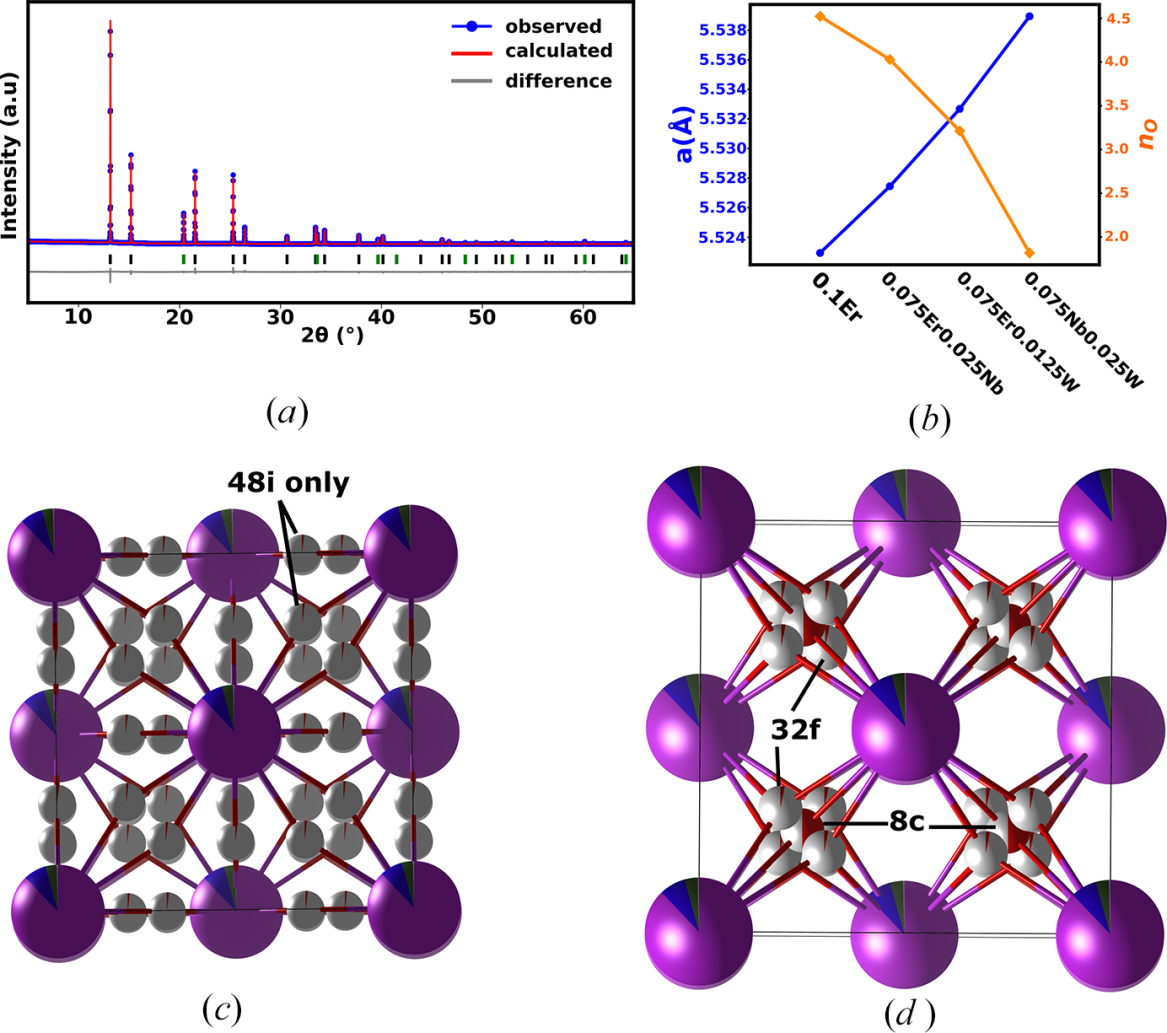
(*a*) Rietveld refinement fit of the 10D5ESB composition (*R*_wp_ = 1.62). Green *hkl* ticks are for the diamond internal standard (*Fd*3*m*). (*b*) The correlation between the unit-cell parameter *a* and the number of oxygens (*n*_O_) in the 8*c* site per unit cell as the concentration of Er is reduced. (*c*) and (*d*) show the position of the O ions in the 48*i* and the 32*f* and 8*c* sites, respectively, relative to the cations. Purple/navy/green wedges represent cations, red wedges represent oxide ions and white wedges represent oxide ion vacancies.

Rietveld refinement results [Fig. 2 ▸(*a*), Tables S1–S8 and Figs. S1–S3] confirmed the average structure to be that of the δ phase and an increase in unit-cell parameter, *a*, as the concentration of Er^3+^ was being reduced [Fig. 2 ▸(*b*)]. As noted by Borowska-Centkowska *et al.* (2020 ▸) and Leszczynska *et al.* (2014 ▸), since W^6+^ and Nb^5+^ are smaller than Er^3+^, they favour a tetrahedral geometry in the structure [even when tetrahedrally coordinated W^6+^ (0.42 Å) and Nb^5+^ (0.48 Å) are smaller than Er^3+^ (0.89 Å) (Shannon, 1976 ▸)]. This results in O atoms occupying more 32*f* and/or 48*i* sites rather than the 8*c* sites around these metal ions as compared to the equivalents for Er^3+^. This leads to an expansion in the unit cell due to the position of 48*i* and 32*f* sites relative to the cations [Figs. 2 ▸(*c*) and 2 ▸(*d*)], counteracting possible contraction due to the reduced average radius of W^6+^ and Nb^5+^ cations. The decrease in the occupancy of the 8*c* sites as Er^3+^ is replaced by W^6+^ and/or Nb^5+^ was observed despite the limited sensitivity of X-rays to oxide ions in the midst of heavy metals like Bi [see Fig. 2 ▸(*b*)]. A similar trend in the occupancy of the 8*c* sites was also observed from neutron diffraction data in a similar system (Leszczynska *et al.*, 2014 ▸).

### 3.2. Total scattering and the PDF technique

**Figure 3:**
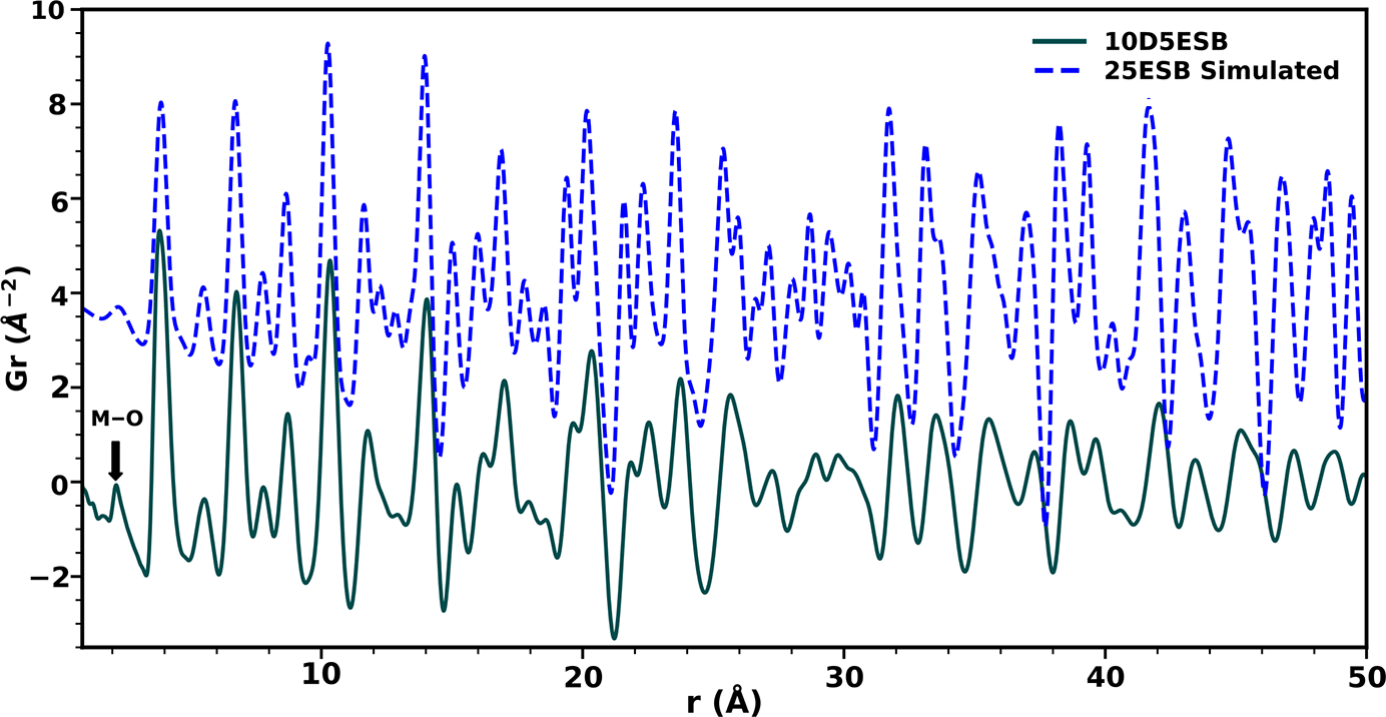
Simulated and experimental reduced total PDFs of 25ESB and 10D5ESB, respectively.

Information such as average bond lengths obtained from peak positions and disorder inferred from peak broadness can be extracted in a model-independent way (*i.e.* without a structural model) from a PDF because of its definition as the measure of the probability of finding an atom at a distance *r* from a reference atom (Egami & Billinge, 2003 ▸). Qualitative analysis of the reduced PDFs of all the systems explored in this work showed that the observed PDFs are similar to the simulated reduced PDF of 25ESB, which has been reported to have the δ phase with space-group type *Fm*3*m* (Fig. 3 ▸) (Battle *et al.*, 1987 ▸). This confirmed the results obtained from the Rietveld refinement. The modal *M*–O bond length obtained from fitting the first peak using *xPDFsuite* (shown by a black arrow in Fig. 3 ▸) varied between 2.15 and 2.16 Å for the systems synthesized (Table 1 ▸). These are in the same range as the modal bond lengths reported by Hull *et al.* (2009 ▸) and Norberg *et al.* (2011 ▸) for the pure α-, β- and δ-Bi_2_O_3_ polymorphs. The *M*–O peak is also highly asymmetric for the synthesized samples, reflecting a non-Gaussian distribution of *M*–O bond lengths in the structure. Comparison of these bond distances with those obtained from Rietveld refinement of Bragg data (Table 1 ▸) of the synthesized samples when the oxygens are predominantly placed in the 8*c* site (which should give modal *M*–O distances) shows some discrepancy and this indicates a difference between the average and local structures as explained in greater detail by Norberg *et al.* (2011 ▸).

**Figure 4:**
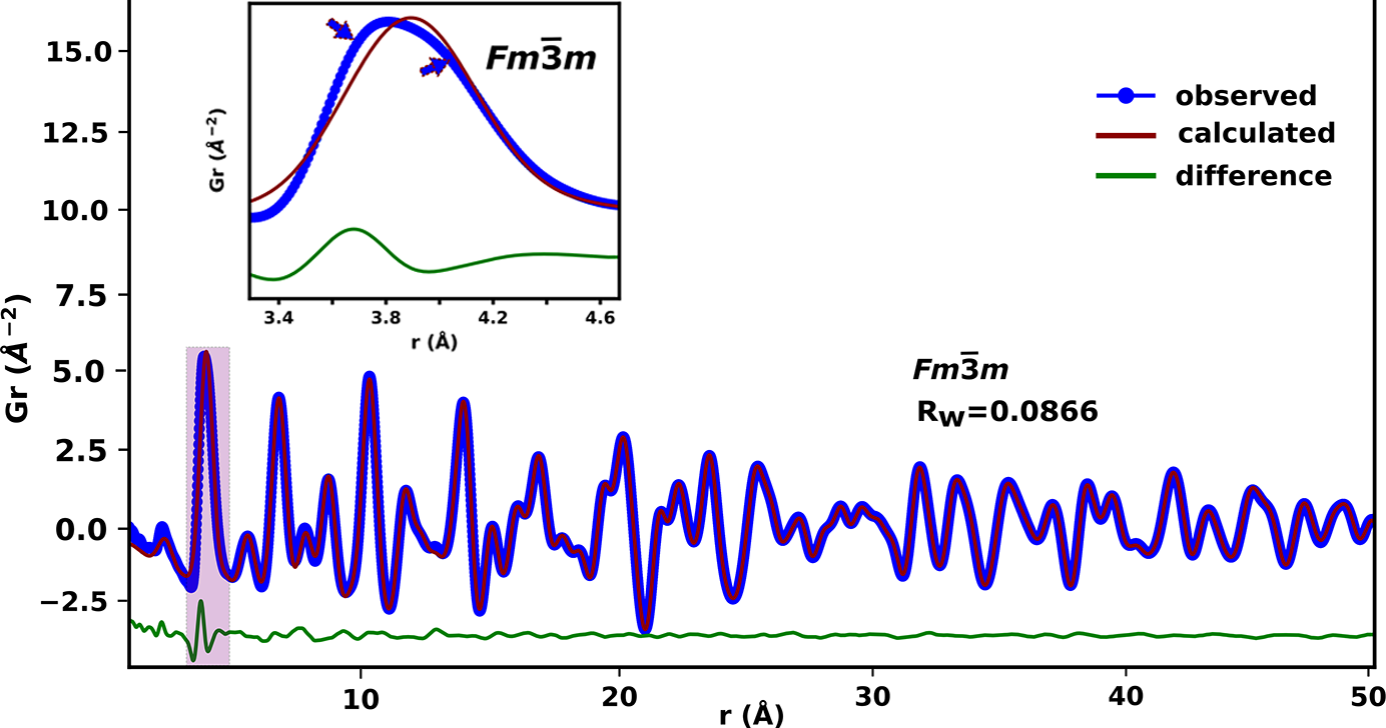
Small-box modelling of the reduced total PDF data of 10D5ESB. The inset and arrows highlight the misfit and asymmetry on the second peak dominated by the shortest *M*–*M* distances.

Small-box modelling of the PDF data of 10D5ESB using the *Fm*3*m* model produced a very good fit in the *r* range 5–50 Å (Fig. 4 ▸). This means that the model describes the intermediate and average structure of the system very well and agrees with the Rietveld refinements results discussed earlier. However, a closer look at the first *M*–*M* peak at around 3.9 Å reveals a poor fit (Fig. 4 ▸, inset), which is due to the presence of a pronounced shoulder and indicates that there really are at least two distinct *M*–*M* distances. Furthermore, the shoulder occurs at higher *r* than the main peak, which cannot be explained by a smaller weighted average *M*–*M* distance expected from the shorter radii of the substituents. The smaller weighted average *M*–*M* distance due to the substituents should cause an asymmetry to appear at lower *r* than the main peak if the substituents were occupying the 4*a* site in *Fm*3*m* as observed by Gateshki *et al.* (2007 ▸). There was also no observed evidence of phase separation in the high-resolution synchrotron Bragg data (see Fig. 1 ▸). This suggested a shift in the metal cations to different positions in the local structure; an observation that cannot be easily captured by a Rietveld refinement.

**Figure 5:**
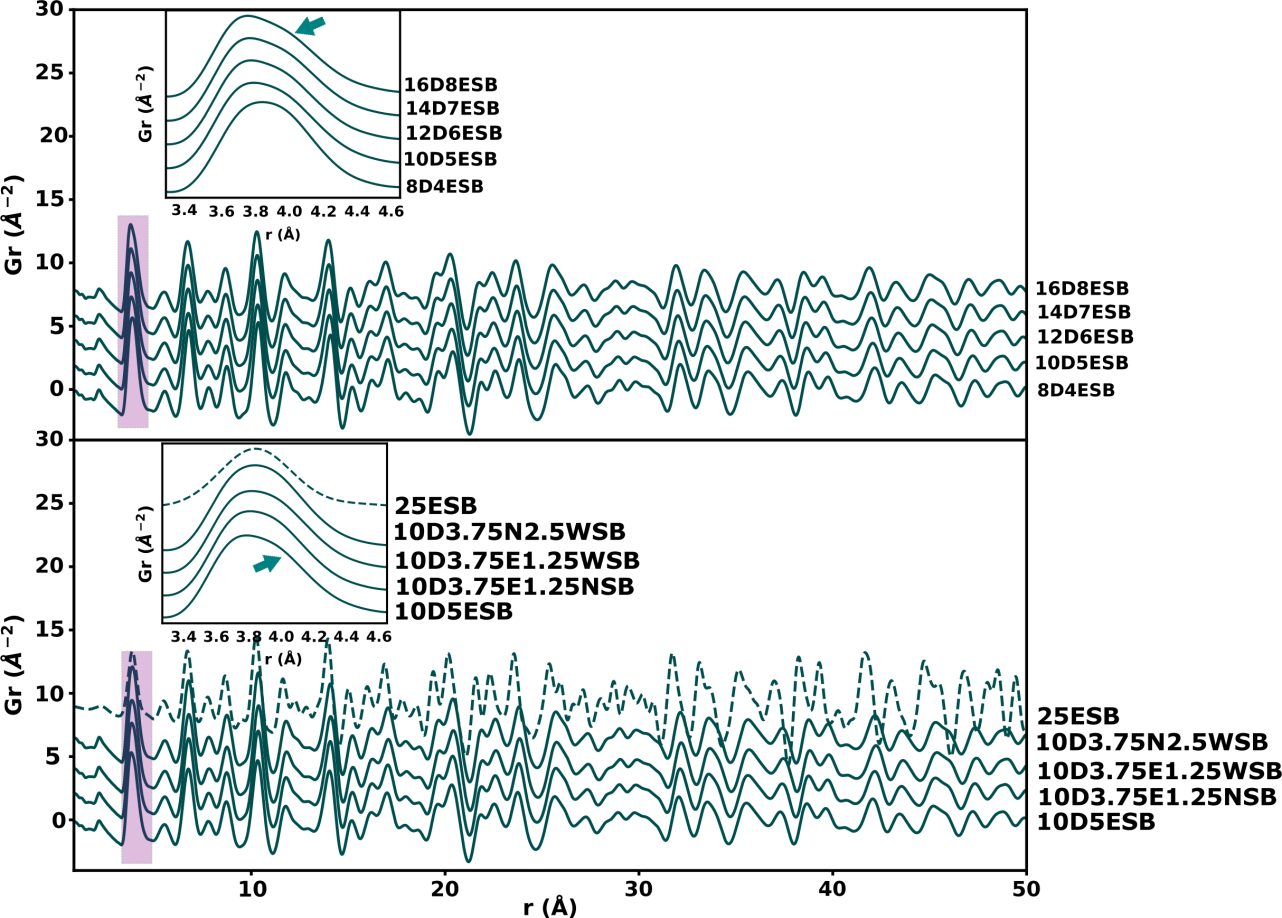
Reduced experimental total PDFs of the four specified systems and that simulated for 25ESB are shown. Evolution of the PDFs as a function of total substituent concentration is also shown. The insets and arrows highlight the shoulders on the second peak dominated by the shortest *M*–*M* distances.

The peak asymmetry and shoulder were amplified with the increase in total substituent concentration, suggesting that the peak asymmetry was induced by the substituents (Fig. 5 ▸). All the PDF were produced using the same parameters (such as *Q*_min_ and *Q*_max_). Interestingly, in the system without the cation Er^3+^, *i.e.* DNWSB, the peak asymmetry is very subtle, but the peak is still broad and is just flatter at the top (Fig. 5 ▸). This suggested that the Er^3+^ cations contribute more to the asymmetry. In the simulated data for 25ESB, there is no peak asymmetry because both the Bi and Er cations were distributed in one site (4*a*) in this structure.

It has been reported that the cations can be displaced along the 〈100〉, 〈110〉 and 〈111〉 directions which correspond to the displacement of cations from 4*a* to the 24*e*, 48*h* and 32*f* sites, respectively (Hull *et al.*, 2009 ▸; Battle *et al.*, 1986 ▸; Battle *et al.*, 1987 ▸). In this work, when some of the Bi^3+^ (50% maximum) and the substituent cations (50% maximum) were placed on these sites while the rest remained at the 4*a* sites in the structure and the models were refined against the data of the 10D5ESB composition, there were no improvements in the fits (Figs. S4–S6). This was observed in all the substituted systems explored. The positions of the main peak (at ∼3.81 Å) in the first *M*–*M* peak (see Fig. 5 ▸, inset) and that of the shoulder (at ∼4.10 Å) are not at the position (∼ 3.91 Å) you would expect for the cell size obtained from Rietveld refinement of the 10D5ESB composition, hence the mismatch in the fit (see Fig. 4 ▸ inset). The intensity of the shoulder is also more than halfway above that of the main peak (Fig. 5 ▸, green arrows in insets). The total substituents concentration and the X-ray atomic form factors are less than those of Bi for all the substituents, therefore a displacement of substituents alone away from 4*a* site is not enough to cause the shoulder. The Bi^3+^ cations should be involved in forming the shoulder and/or asymmetry.

**Figure 6:**
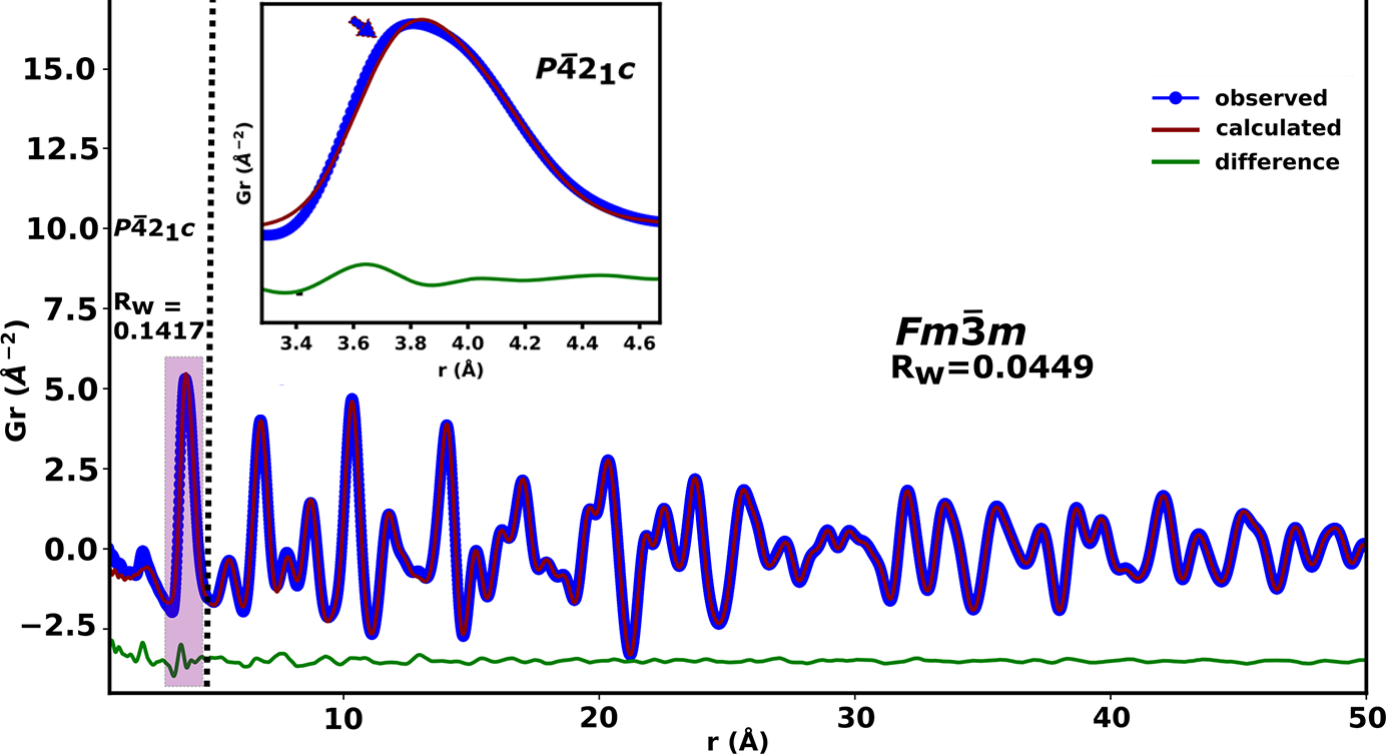
Small-box modelling of the reduced total PDF data of 10D5ESB illustrating how the *P*42_1_*c* model reproduces the asymmetry on the first *M*–*M* peak.

The model for the β–Bi_2_O_3_ phase (*P*42_1_*c*) was also tested at the local length scale (*r* ≤ 4.5 Å, Fig. 6 ▸) in 10D5ESB since the cation sublattice of this phase is essentially similar to that of the δ-Bi_2_O_3_ phase. The *P*42_1_*c* model fitted the data better than the *Fm*3*m* model (see Fig. 4 ▸ and compare with Fig. 6 ▸) in the range *r* ≤ 4.5 Å but does not give a shoulder. Therefore, both these models are not satisfactory in describing the overall local structure of the metal sublattice and the first *M*–O peak on their own for all the substituted systems explored.

**Figure 7:**
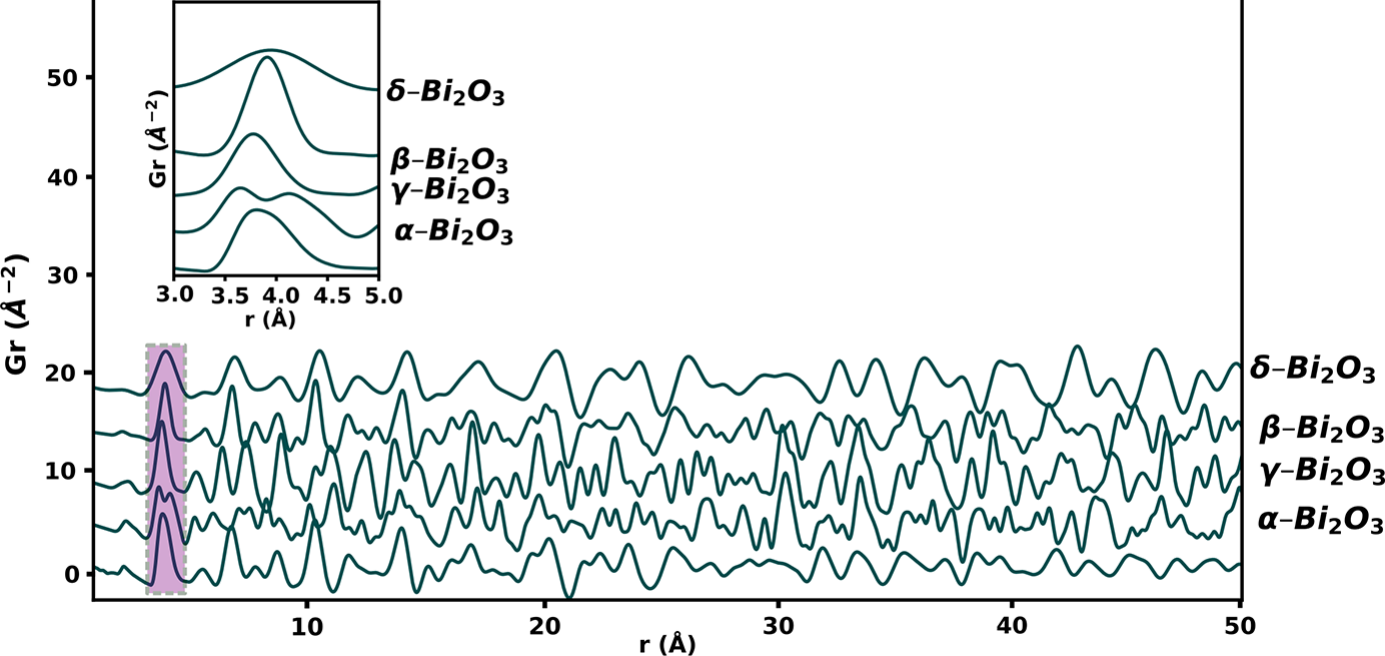
Simulated reduced total PDFs of the different polymorphs of Bi_2_O_3_.

A plot of the simulated PDFs of the four main unsubstituted polymorphs of Bi_2_O_3_ revealed that the model structures with space group types *Fm*3*m* and *P*42_1_*c* do not exhibit any shouldering in the second peaks (Fig. 7 ▸, inset), and that second peaks are at a slightly higher *r* position than that obtained from the observed data of the substituted systems. The model of the γ-Bi_2_O_3_ phase (*I*23) has its first *M*–*M* peak at a similar position to that of the first *M*–*M* peak in the observed data of 10D5ESB but does not display any asymmetry or a shoulder.

**Figure 8:**
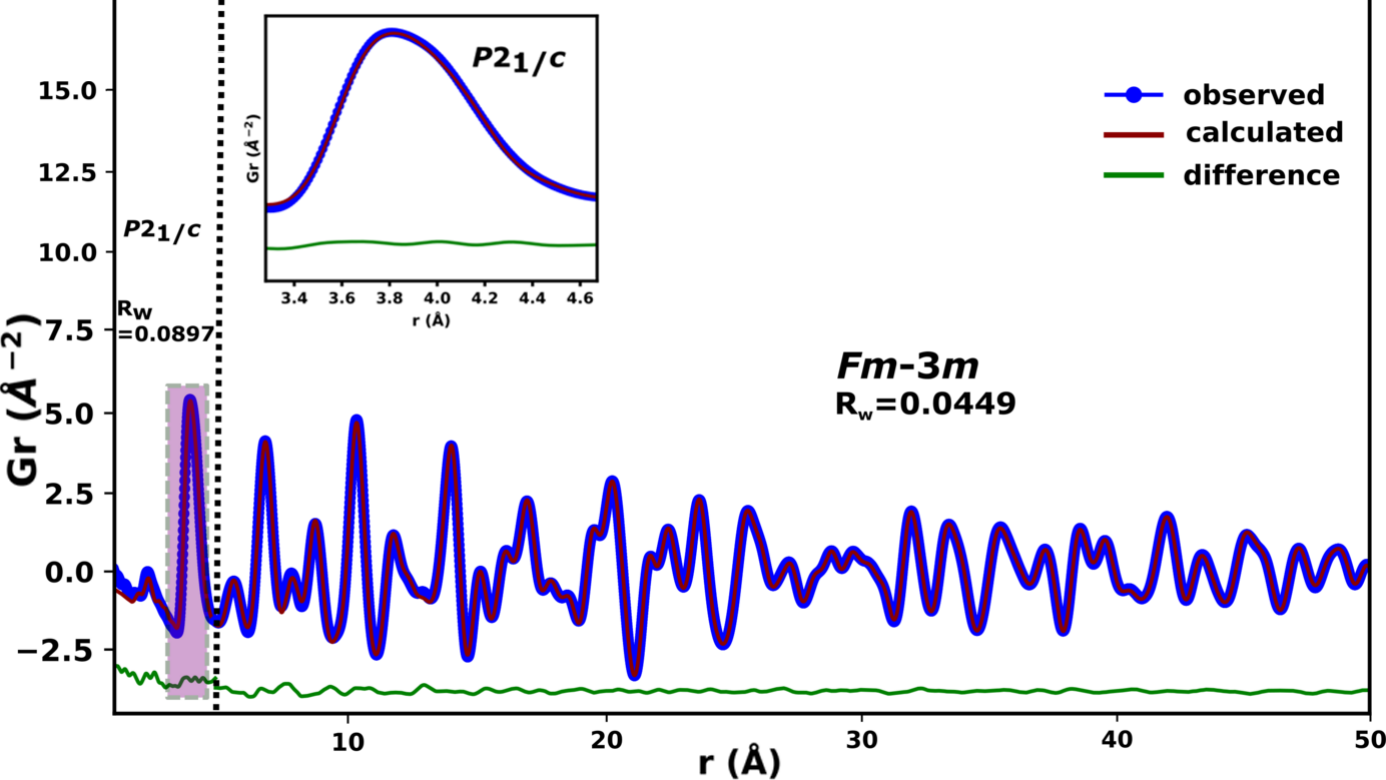
Small-box modelling of the reduced total PDF data of 10D5ESB illustrating how the *P*2_1_/*c* model fits the data at low *r*.

The *P*2_1_/*c* model of the monoclinic α-Bi_2_O_3_ phase show some similarity to the observed data of 10D5ESB (Fig. 8 ▸, inset). The shoulder and asymmetry of the first *M*–*M* peak and the direction of tailing are similar. However, the peak positions are not closely matched to that for the substituted sample. The highly asymmetric first peak at ∼2.15 Å (predominantly from the *M*–O correlations) for the α-Bi_2_O_3_ polymorph also reflects that seen for the substituted systems. A similar situation was reported by Gateshki *et al.* (2006 ▸) for zirconium oxide involving the monoclinic (*P*2_1_/*c*) and cubic (*Fm*3*m*) phase.

Small-box modelling using *P*2_1_/*c* at the local level (up to 4.5 Å) produced a very good fit (Fig. 8 ▸, inset). This is a very short range and thus the parameters are highly correlated, but this does suggest that the substituents induced some local level monoclinic-like distortions to stabilize the δ-Bi_2_O_3_-like phase to ambient temperature; a phenomenon common in materials with the fluorite structure (Gateshki *et al.*, 2007 ▸; Ishizawa *et al.*, 1999 ▸). This is also a common strategy for fitting and showing local level distortions in PDF (Gateshki *et al.*, 2007 ▸; Sardar *et al.*, 2010 ▸; Scavini *et al.*, 2012 ▸; Mamontov *et al.*, 2003 ▸). Fitted parameters from the PDFs are summarized in Tables S9–S16. It should be noted that the average structure of the α-Bi_2_O_3_ phase is different from both the observed data of the substituted systems and the δ-Bi_2_O_3_ phase (see Fig. 7 ▸, beyond 4.5 Å). These results demonstrate the inadequacy of traditional crystallographic methods in fully describing the structure of some functional materials and reinforce the idea of using both the local and average structure to describe the structure of such materials more comprehensively.

### 3.3. The *L*_3_ edge XANES analyses of Dy, Er and W dopants

Total atomic X-ray PDF can have some limitations as a local structure probe. Peaks from different atomic pairs with comparable interatomic distances can overlap and make it hard to obtain an accurate picture of the local environment around a particular type of atom. This limitation is amplified when the weighted X-ray atomic form factors of the atomic pairs with overlapping peaks are comparable (*e.g.* Dy–O and Er–O). When lighter atoms are in the midst of heavier ones, the signals from the heavier atoms dominate and sensitivity of the technique to the lighter atoms is significantly reduced as is the case of O in the presence of Bi, Dy and W in this work. To probe the local environment of a particular substituent, an element-specific technique such as XAS can provide insights into the electronic structure, oxidation state and geometry of the site occupied by a specific absorber. In this work, XANES data of Dy, Er and W were used to provide some insights into the geometry of the sites occupied by the substituent in the materials.

**Figure 9:**
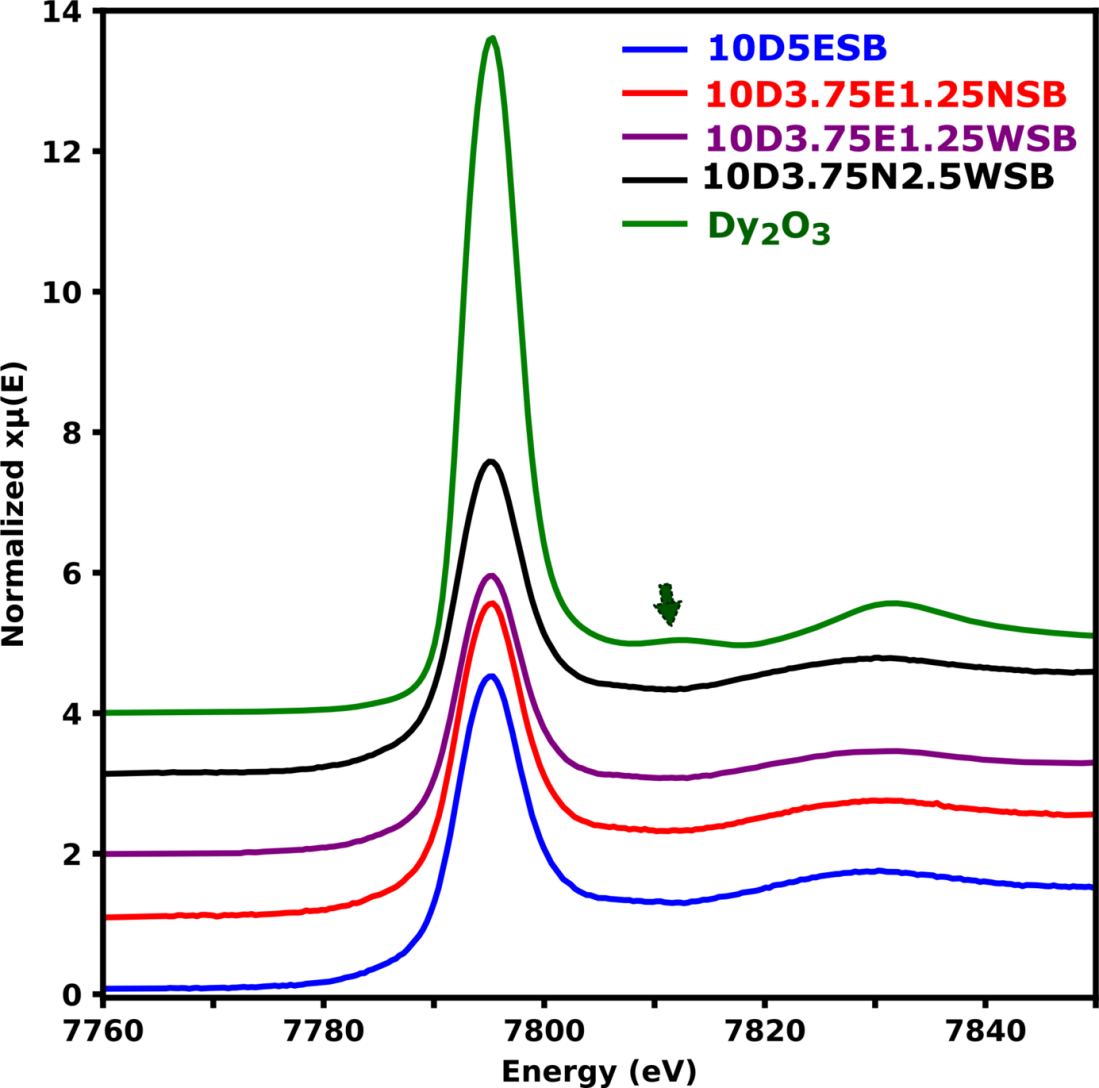
Normalized spectra of the Dy *L*_3_ edge. The arrow indicates the peak characteristic of the *C*-type (*Ia*3) structure of Dy_2_O_3_.

The normalized spectra of the Dy *L*_3_ edge XANES in all four systems and the pure Dy_2_O_3_ oxide are shown in Fig. 9 ▸. The white line (intense peak) feature in the Dy *L*_3_ edge XANES spectra [as well as the Er *L*_3_ edge XANES spectrum in Fig. S7(*a*)] is attributed predominantly to the promotion of an electron from 2*p*_3/2_ to 5*d* orbitals and all the spectra showed similar characteristics. The Dy_2_O_3_ powder was included as a reference to probe the oxidation state of Dy in the bis­muth oxide solid solutions. The peak at approximately 7810 eV indicated with an arrow in Fig. 9 ▸ is characteristic of Dy_2_O_3_ and is associated with a well ordered average structure (Tiwari *et al.*, 2017 ▸). It was virtually absent in the spectra of the δ phases, suggesting that the Dy cations were successfully incorporated into the disordered Bi_2_O_3_ average structure.

**Figure 10:**
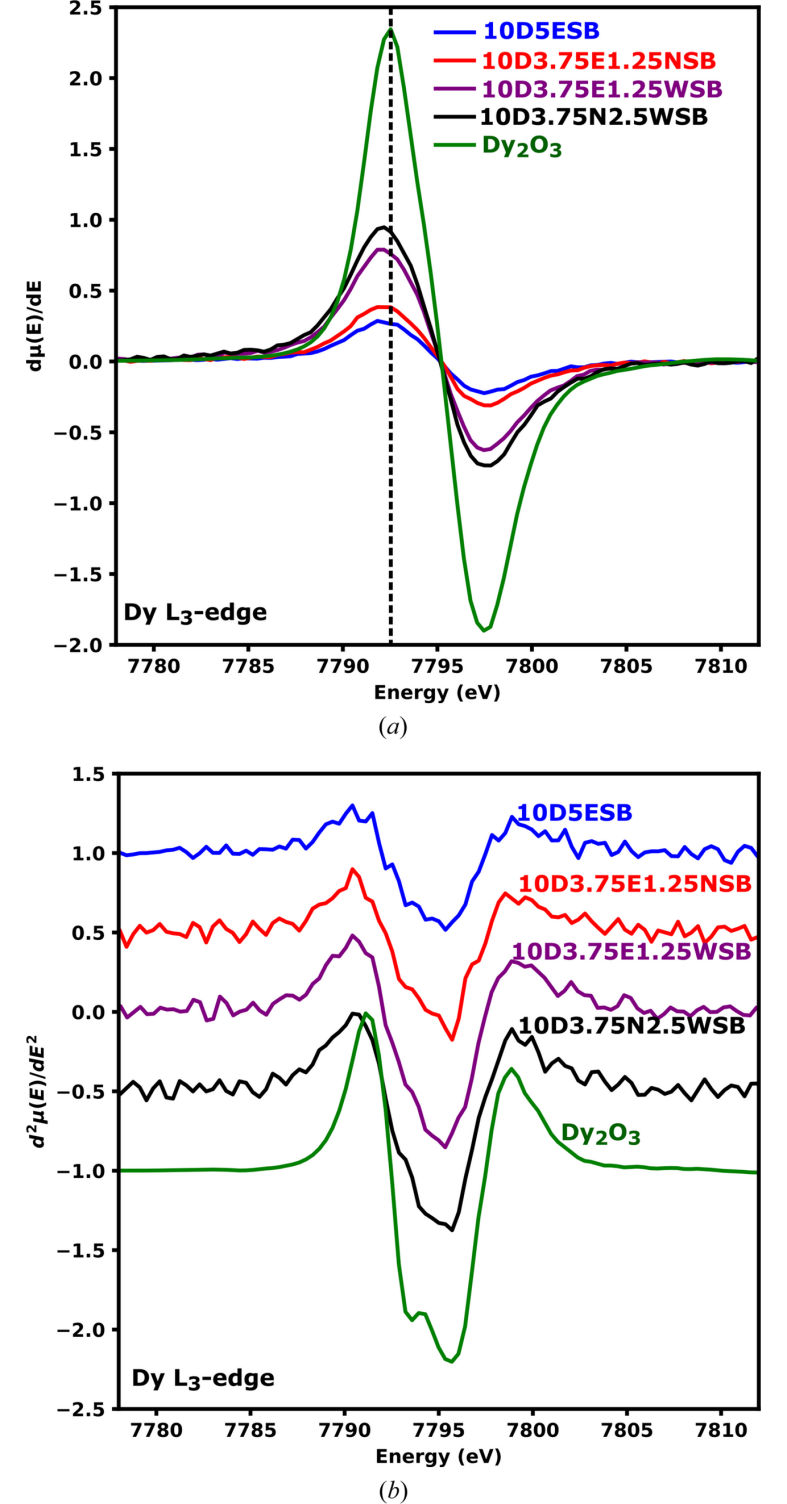
Normalized first (*a*) and second (*b*) derivative Dy *L*_3_ edge XANES spectra of the Dy containing compositions.

The first-derivative normalized spectra showed virtually the same edge position – taken as the position of the highest point in the plot [Fig. 10 ▸(*a*), dashed line]. The edge position is heavily dependent on the oxidation state of the absorber and the local environment around it. This unsurprisingly suggests that the Dy has a similar oxidation state across all compositions including in Dy_2_O_3_ (Das *et al.*, 2018 ▸). To better understand the electronic structure of Dy, the second derivative of the white lines of the substituted systems were considered, as it is commonly done for most rare earth elements (Ishii *et al.*, 1999 ▸; Anjana *et al.*, 2018 ▸; Asakura *et al.*, 2015 ▸), and these were compared with those of the parent oxide (Dy_2_O_3_). Dy_2_O_3_ showed two distinct minima in the second-derivative spectrum [Fig. 10 ▸(*b*)] indicating that there are at least two different electronic transitions giving rise to the observed white line. With Dy_2_O_3_ having the space group type *Ia*3 (Fig. S8), the Dy cations occupy two sites; namely, the 8*b* site which has *C*_3i_ site symmetry and the 24*d* site with a site symmetry of *C*_2_ (Fig. S8, inset). The 5*d* states will be split differently in these sites. Therefore, the overall XAS spectrum of Dy_2_O_3_ should at least reflect the weighted average from the two sites occupied by the cations. From the crystallographic information file for Dy_2_O_3_ (ICSD No. 66736), each site has a full occupancy of one, therefore, at most, the ratio of the contribution to the spectra of the 8*b* and 24*d* sites is expected to be 1:3.

It has been reported that for the Dy and Er cations in a pure O_h_ environment, essentially, a single minimum is obtained from the second derivative of the spectra elements (Ishii *et al.*, 1999). This would be expected from the spectra of Dy^3+^ and Er^3+^ at the 4*a* sites in the average structure of δ-Bi_2_O_3_ which has O_h_ site symmetry. However, the overall shape of the second derivatives of the spectra obtained from the compositions with Dy^3+^ [Fig. 10 ▸(*b*)] and Er^3+^ [Fig. S7(*b*)] resembles that of Dy^3+^ in Dy_2_O_3_. This is an indication that at least some of the Dy^3+^ and Er^3+^ cations are not occupying the 4*a* sites in the *Fm*3*m* but have local bonding environment similar to those found in Dy_2_O_3_ or Er_2_O_3_. This is more pronounced for the Nb-containing compositions. The smaller peak minima on the left side of the second derivatives were not prominent in the substituted bis­muth oxide compositions compared to the parent oxide (Dy_2_O_3_) but still noticeable and comparable. This was probably due to a slightly lower ligand field splitting energy of the 5*d* orbitals in the structure and the slight changes in the site symmetries of Dy^3+^ and Er^3+^ in the substituted compositions compared to the environments of Dy^3+^ and Er^3+^ in the pure oxides [Figs. 10 ▸(*b*) and S7(*b*)]. This is also corroborated by the comparable energy values of the Gaussian peak positions obtained from fitting the white lines of Dy_2_O_3_ spectrum and those of the substituted compositions (Figs. S8 and S9). The energy difference from the split peaks ranges between 1.88 and 2.21 eV. The ligand field splitting energy for Dy_2_O_3_ could not be found in the literature but these values are closer to the splitting energy caused by ligand field (2.9–3.37 eV) reported by Anjana *et al.* (2018 ▸) for Yb_2_O_3_ and lower than the 7 eV caused by oxidation change of Yb.

**Figure 11:**
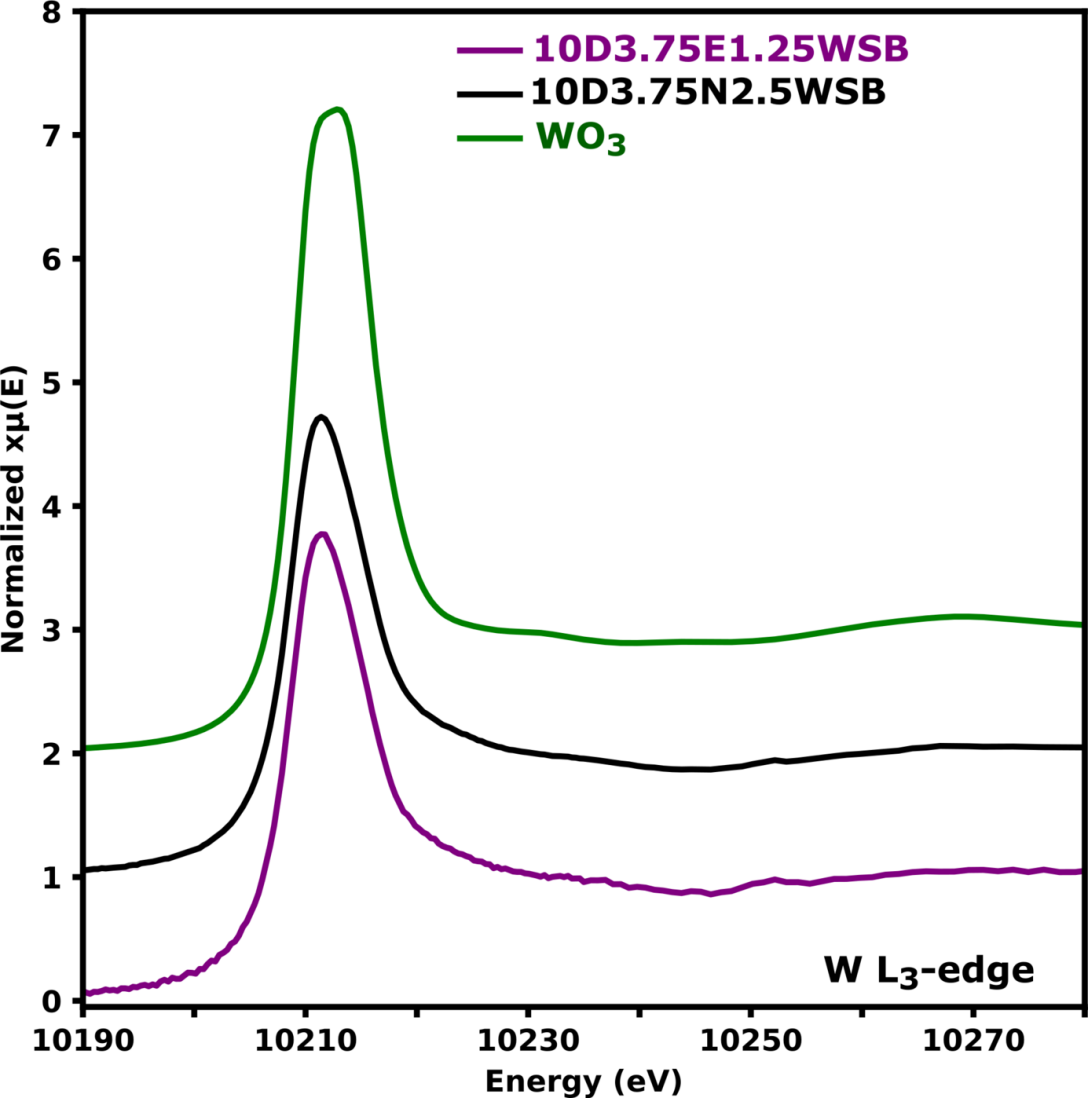
Normalized W *L*_3_ edge XANES spectra of WO_3_, 10D3.75E1.25WSB and 10D3.75N2.5WSB.

The study of the W *L*_3_ edge XANES was conducted to gain insight into the coordination environment and oxidation state in W-doped bis­muth oxide solid solutions. These spectra correspond to the electronic transition from the 2*p*_3/2_ to the empty 5*d* orbitals of W. The normalized XANES spectra are provided in Fig. 11 ▸.

**Figure 12:**
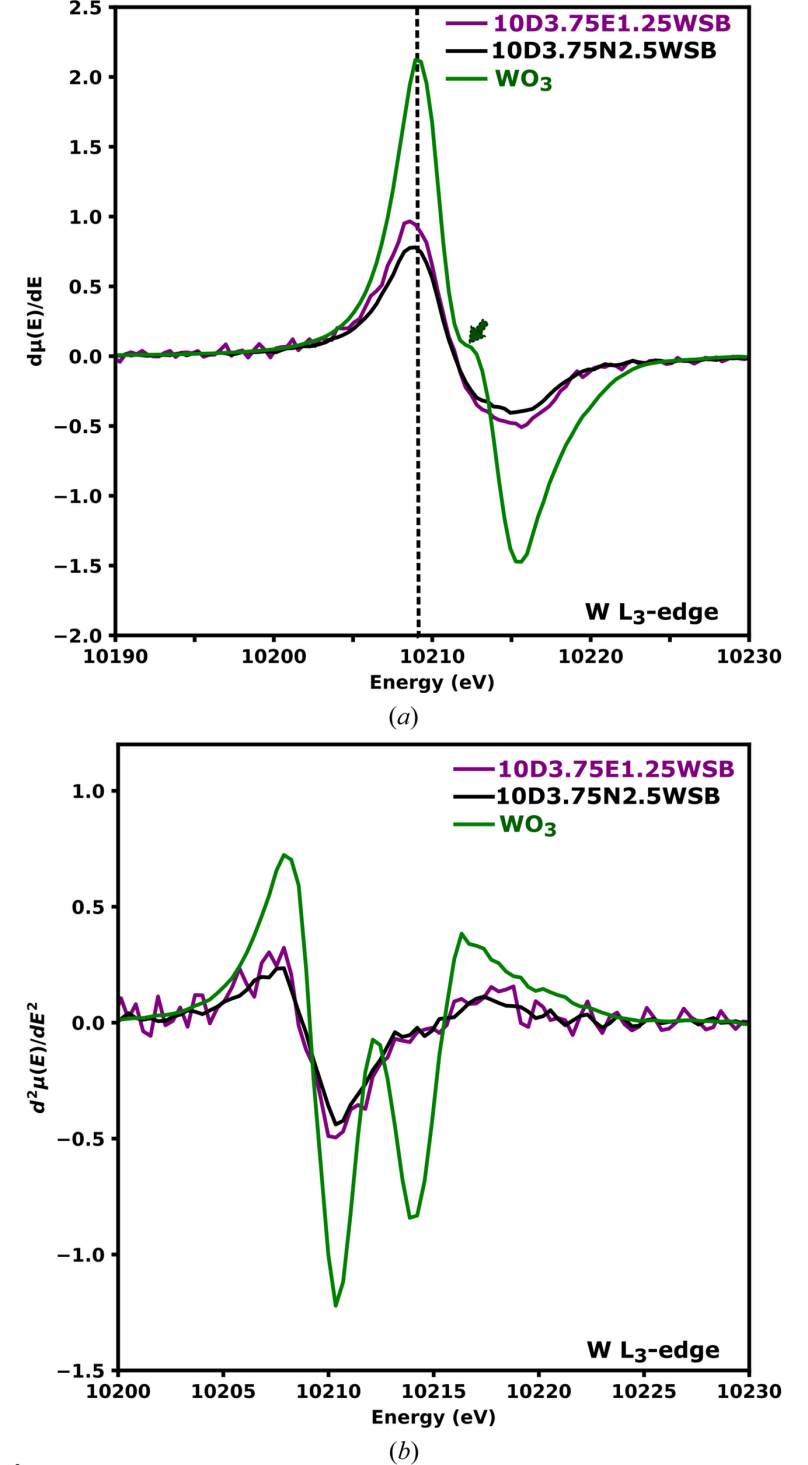
Normalized first (*a*) and second (*b*) derivative W *L*_3_ edge XANES spectra of 10D3.75E1.25WSB, 10D3.75N2.5WSB and WO_3_. The arrow in (*a*) indicates an inflection point characteristic of six-coordinated W^6+^ species.

The WO_3_ spectrum is consistent with that from literature (Yamazoe *et al.*, 2008 ▸); the broad feature in the white line of octahedrally coordinated W is attributed to the relatively large crystal field splitting of the *d*-orbitals and is large enough to produce two distinct peaks, each corresponding to the *t*_2_ and *e* set of orbitals, respectively. However, in the 10D3.75E1.25WSB and 10D3.75N2.5WSB compositions, the white line peaks are more asymmetric and narrower, showing no clear distinction of the 5*d* orbitals splitting. These results suggest that in these formulations W might be tetrahedrally coordinated, since the crystal field splitting between the *e* and *t*_2_ sets of orbitals in tetrahedrally coordinated W is relatively smaller which would lead to narrower white lines for these compositions. From the normalized first-derivative spectra [Fig. 12 ▸(*a*)] it was seen that the relatively smaller ligand field splitting in tetrahedrally coordinated W results in derivate spectra that have peaks from transitions to *e* and *t*_2_ that cannot be deconvoluted hence the absence of a pronounced inflection point and a sharper white line. The small inflection at approximately 10211 eV shown by the green arrow in Fig. 12 ▸(*a*) is an indicator of octahedral coordination around W for the WO_3_ powder [Fig. 12 ▸(*a*)] as measured in this work and is also reported by Wind *et al.* (2017 ▸). For the 10D3.75E1.25WSB and 10D3.75N2.5WSB compositions, the small inflection is undiscernible at 10212 eV. This further suggests that these compositions might contain predominantly tetrahedral W environments as observed by Wind *et al.* (2017 ▸) and also alluded to by the expansion of the unit cell in the Bragg data without a change in average structure.

The second derivative of the XANES spectrum of WO_3_ shows well resolved minima ∼3.52 eV apart [Fig. 12 ▸(*b*)] and WO_3_ has distorted octahedra units as shown in the inset of Fig. S11. From curve fitting as shown in the supplementary information in Fig. S11 (energy gap of the first two peaks), the estimated value of ligand field stabilization energy (Δ_O_) of WO_3_ and substituted compositions was found to be ∼3.52 eV. This is very similar to the ∼3.7 eV found by Yamazoe *et al.* (2008 ▸) for WO_3_. However, for the 10D3.75E1.25WSB and 10D3.75N2.5WSB formulations, the second minimum at higher energies are far less pronounced, with only significant asymmetry discernible. This has been attributed to the presence of the mixture of octahedral coordination and a predominantly tetrahedral coordination environment in W-containing materials (Yamazoe *et al.*, 2008 ▸). To the authors knowledge, this is the first spectroscopic evidence showing that W in some δ phases occupy predominantly tetrahedrally coordinated environments. This is further evidence that the average structure is not a comprehensive description of the structure of these materials.

## 4. Conclusions

In this work, high-resolution synchrotron Bragg and PDF data were used to confirm the stabilization of δ phases to ambient temperature using a minimum total substituent concentration in the range 15–16.25 mol% in ternary and quaternary oxides of Bi_2_O_3_. It was observed that when Er^3+^ substituent cations were replaced with W^6+^ and/or Nb^5+^, the unit-cell parameter *a* counterintuitively expanded and this was correlated to a decrease in the occupancy of the oxide ions in the 8*c* sites. It was also observed from total scattering and XAS data that the substituent cations are not all replacing Bi at the 4*a* site in the stabilized δ phases, but instead they seem to induce local monoclinic-like distortions in the metal sublattice. This effect was more pronounced in the system with Er and Dy cations only and seemed to increase with increase in Er concentration. X-ray absorption spectroscopy showed that the local structure around the Dy^3+^ cations in the substituted systems was similar to that of Dy^3+^ in Dy_2_O_3_, which is different from the site geometry of metal cations in the average structure of δ-Bi_2_O_3_. X-ray absorption spectroscopy also indicated that the W cations are predominantly tetrahedrally coordinated in the stabilized δ phases (therefore predominantly not in the 4*a* site), which is likely a contributing factor to the lattice expansion of compositions where W cations were replacing Er and pushing more oxide ions into interstitial (48*i*) or interstitial-like (32*f*) sites. This highlights the role of the substituents cations in influencing the local structure of the metal sublattice of the host and warrants further investigation using advanced methods such as big-box modelling.

## 5. Related literature

The following references are cited in the supporting information: Antic *et al.* (1993 ▸); Diehl & Brand (1978 ▸); Vogt *et al.* (2013 ▸).

## Supplementary Material

pdCIF file for sample with composition corresponding to Bi1.7Dy0.2Er0.1O3. DOI: 10.1107/S2052520625009400/ra5156sup1.txt

pdCIF file for sample with composition corresponding to Bi1.7Dy0.2Er0.075W0.0125O3. DOI: 10.1107/S2052520625009400/ra5156sup2.txt

pdCIF file for sample with composition corresponding to Bi1.7Dy0.2Er0.075Nb0.025O3.025. DOI: 10.1107/S2052520625009400/ra5156sup3.txt

pdCIF file for sample with composition corresponding to Bi1.7Dy0.2Nb0.075W0.025O3.075. DOI: 10.1107/S2052520625009400/ra5156sup4.txt

Tables S1-S21 and Figs. S1-S12. DOI: 10.1107/S2052520625009400/ra5156sup5.pdf

## Figures and Tables

**Table 1:** Modal bond lengths (Å) obtained from both PDF peak fitting and Rietveld refinement of Bragg data for a given composition

System	PDF	Rietveld
10D5ESB	2.15	2.39
10D3.75E1.25WSB	2.15	2.40
10D3.75E1.25NSB	2.16	2.39
10D3.75N2.5WSB	2.16	2.40

## Data Availability

Raw data are available upon request.
