# Supplementary material for: Probing the local structure of Bi2O3 chemical derivatives: the neglected cation sublattice
Source: Acta Crystallogr B Struct Sci Cryst Eng Mater. 2025 Nov 24;81(Pt 6):595–604. doi: 10.1107/S2052520625009400 (PMC12786384; doi:10.1107/S2052520625009400)
Supplement: Supplementary file 5 [file b-81-00595-sup5.pdf]

STRUCTURAL SCIENCE
CRYSTAL ENGINEERING
MATERIALS

Volume 81 (2025)

Supporting information for article:

Probing the local structure of Bi2O3 chemical derivatives: the neglected cation sublattice

Sikhumbuzo M. Masina, Gugulethu C. Nkala, Kevin H. Stone, Daniel Olds, Caren Billing and David G. Billing

Table S1 Crystallographic data and refinement parameters for the $Bi_{1.7}Dy_{0.2}Er_{0.1}O_3$ composition at ambient temperatures.

Composition	$Bi_{1.7}Dy_{0.2}Er_{0.1}O_3$
R_{wp} (%)	1.61
Formular weight	906.210
Space group	*Fm*-3*m*
a /Å	5.522934(3)
Cell volume /$Å^3$	169.358
Z	2
Crystal density /$g.cm^{-3}$	8.92030

Table S2 Refined structural parameters for the $Bi_{1.7}Dy_{0.2}Er_{0.1}O_3$ composition at ambient temperatures.

Atom	Site	x	y	z	*Occ*	B_{iso} ($Å^2$)
Bi	$4a$	0	0	0	0.85	2.660(7)
Dy	$4a$	0	0	0	0.10	2.660(7)
Er	$4a$	0	0	0	0.05	2.660(7)
O(1)	$8c$	0.25	0.25	0.25	0.56(3)	6.48(17)
O(2)	$32f$	0.350(7)	0.350(7)	0.350(7)	0.046(7)	6.48(17)

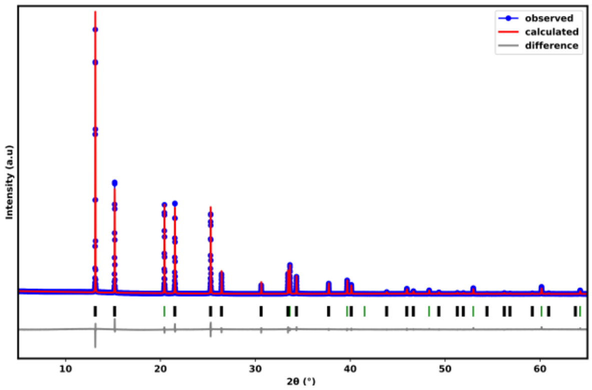

Figure S1 Rietveld refinement for the $Bi_{1.7}Dy_{0.2}Er_{0.075}W_{0.0125}O_3$ composition at ambient temperatures. Green and black hkls are for the diamond internal standard and δ-phase, respectively, with the model modified from Hull (Hull et al., 2009) and Leszczynska (Leszczynska et al., 2013)..

Table S3 Crystallographic data and refinement parameters for the $Bi_{1.7}Dy_{0.2}Er_{0.075}W_{0.0125}O_3$ composition at ambient temperatures.

Composition	$Bi_{1.7}Dy_{0.2}Er_{0.075}W_{0.0125}O_3$
R_{wp} (%)	2.74
Formular weight	906.210
Space group	*Fm*-3*m*
a /Å	5.532680(5)
Cell volume /$Å^3$	169.359(4)
Z	2
Crystal density /$g.cm^{-3}$	8.88529(3)

Table S4 Refined structural parameters for the $Bi_{1.7}Dy_{0.2}Er_{0.075}W_{0.0125}O_3$ composition at ambient temperatures.

Atom	Site	*x*	*y*	*z*	*Occ*	B_{iso} ($Å^2$)
Bi	4*a*	0	0	0	0.8556	2.63(1)
Dy	4*a*	0	0	0	0.1007	2.63(1)
Er	4*a*	0	0	0	0.0378	2.63(1)
W	4*a*	0	0	0	0.0062	2.63(1)
O(1)	8*c*	0.25	0.25	0.25	0.40(3)	4.27(77)
O(2)	32*f*	0.350(4)	0.350(4)	0.350(4)	0.087(6)	4.27(77)

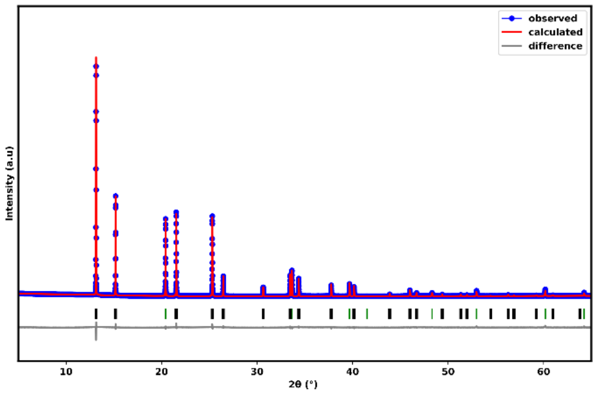

Figure S2 Rietveld refinement for the $Bi_{1.7}Dy_{0.2}Er_{0.075}Nb_{0.025}O_{3.025}$ composition at ambient temperatures. Green and black hkls are for the diamond internal standard and δ-phase, respectively, with the model modified from Hull (Hull et al., 2009) and Leszczynska (Leszczynska et al., 2013).

Table S5 Crystallographic data and refinement parameters for the $Bi_{1.7}Dy_{0.2}Er_{0.075}Nb_{0.025}O_{3.025}$ composition at ambient temperatures.

Composition	$Bi_{1.7}Dy_{0.2}Er_{0.075}Nb_{0.025}O_{3.025}$
R_{wp} (%)	1.70
Formular weight	902.064
Space group	*Fm*-3*m*
a /Å	5.527446(3)
Cell volume /Å^3	168.878
Z	2
Crystal density /g.cm^{-3}	8.86979

Table S6 Refined structural parameters for the $Bi_{1.7}Dy_{0.2}Er_{0.075}Nb_{0.025}O_{3.025}$ composition at ambient temperatures.

Atom	Site	*x*	*y*	*z*	*Occ*	B_{iso} (Å^2)
Bi	4*a*	0	0	0	0.85	2.47(1)
Dy	4*a*	0	0	0	0.10	2.47(1)
Er	4*a*	0	0	0	0.0375	2.47(1)
Nb	4*a*	0	0	0	0.0125	2.47(1)
O(1)	8*c*	0.25	0.25	0.25	0.50(3)	5.75(64)
O(2)	32*f*	0.35(1)	0.35(1)	0.35(1)	0.034(8)	5.75(64)
O(3)	48*i*	0.5	0.195(20)	0.195(20)	0.019(6)	5.75(64)

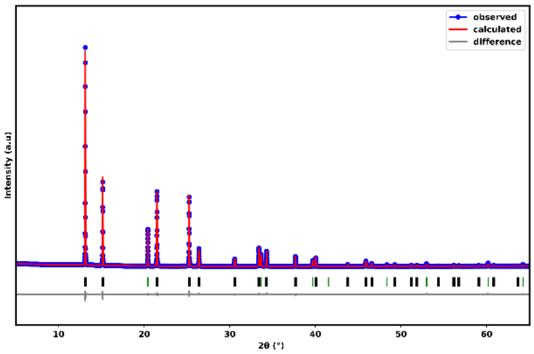

Figure S3 Rietveld refinement for the $Bi_{1.7}Dy_{0.2}Nb_{0.075}W_{0.025}O_{3.075}$ composition at ambient temperatures. Green and black hkls are for the diamond internal standard and δ-phase, respectively, with the δ-phase model modified from Hull (Hull et al., 2009) and Leszczynska (Leszczynska et al., 2013).

Table S7 Crystallographic data and refinement parameters for the $Bi_{1.7}Dy_{0.2}Nb_{0.075}W_{0.025}O_{3.075}$ composition at ambient temperatures.

Composition	$Bi_{1.7}Dy_{0.2}Nb_{0.075}W_{0.025}O_{3.075}$
R_{wp} (%)	1.55
Formular weight	898.258
Space group	*Fm*-3*m*
a /Å	5.538943(3)
Cell volume /$Å^3$	169.934
Z	2
Crystal density /$g.cm^{-3}$	8.77747

Table S8 Refined structural parameters for the $Bi_{1.7}Dy_{0.2}Nb_{0.075}W_{0.025}O_{3.075}$ composition at ambient temperatures.

Atom	Site	*x*	*y*	*z*	*Occ*	B_{iso} ($Å^2$)
Bi	4*a*	0	0	0	0.85	2.430(7)
Dy	4*a*	0	0	0	0.10	2.430(7)
Nb	4*a*	0	0	0	0.0375	2.430(7)
W	4*a*	0	0	0	0.0125	2.430(7)
O(1)	8*c*	0.25	0.25	0.25	0.23(4)	4.91(26)
O(2)	32*f*	0.318(3)	0.318(3)	0.318(3)	0.137(8)	4.91(26)

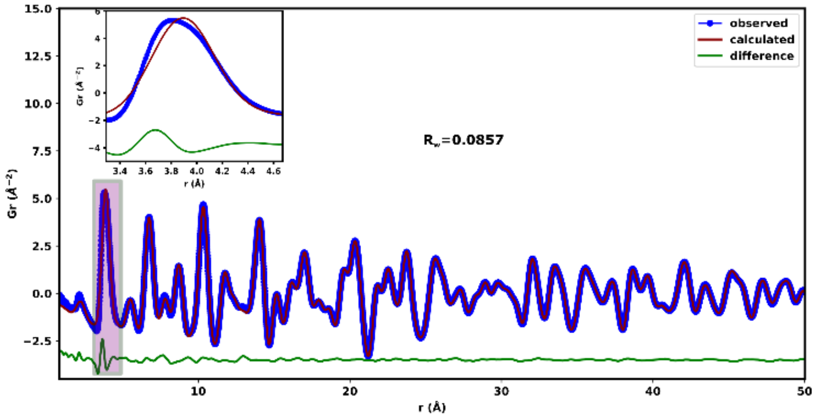

Figure S4 Small-box modelling of the PDF data collected for the $Bi_{1.7}Dy_{0.2}Er_{0.1}O_3$ composition at ambient temperature. This shows how moving half of each type of the metal cations to 24*e* does not improve the fit on the first M-M peak.

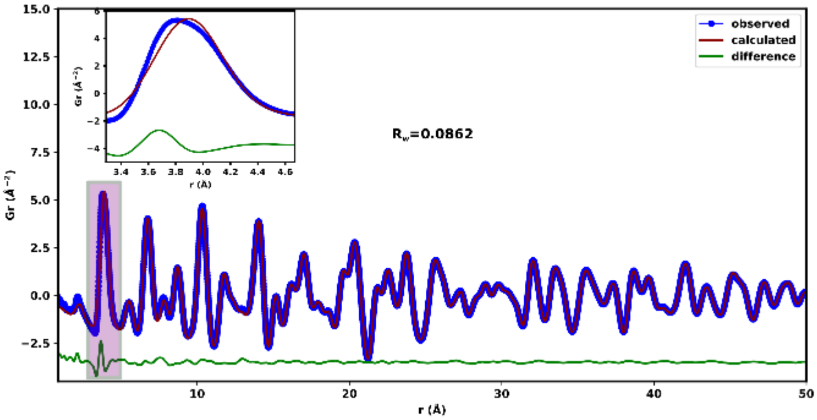

Figure S5 Small-box modelling of the PDF data collected for the $Bi_{1.7}Dy_{0.2}Er_{0.1}O_3$ composition at ambient temperature. This shows how moving half of each type of the metal cations to 48*h* does not improve the fit on the first M-M peak.

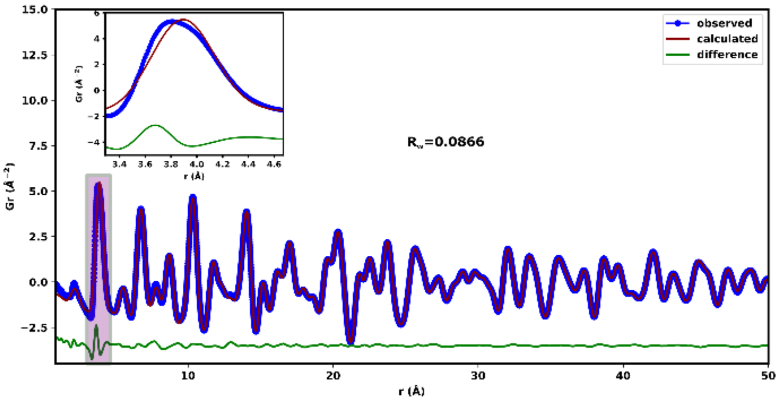

Figure S6 Small-box modelling of the PDF data collected for the $Bi_{1.7}Dy_{0.2}Er_{0.1}O_3$ composition at ambient temperature. This shows how moving half of each type of the metal cations to 32*f* does not improve the fit on the first M-M peak.

Table S9 Crystallographic data and refinement parameters for the $Bi_{1.7}Dy_{0.2}Er_{0.1}O_3$ composition at ambient temperatures from PDF fitting. Fitting range 4.5-50 Å.

Composition	$Bi_{1.7}Dy_{0.2}Er_{0.1}O_3$
R_w (%)	4.6
Formular weight	906.210
Space group	*Fm*-3*m*
a /Å	5.528323(4)
Cell volume /$Å^3$	168.959(4)
Z	2
Crystal density /$g.cm^{-3}$	8.8942(2)

Table S10 Refined structural parameters for the $Bi_{1.7}Dy_{0.2}Er_{0.1}O_3$ composition at ambient temperatures from PDF fitting.

Atom	Site	*x*	*y*	*z*	*Occ*	B_{iso} ($Å^2$)
Bi	4*a*	0	0	0	0.85	3.411 (7)
Dy	4*a*	0	0	0	0.10	3.411 (7)
Er	4*a*	0	0	0	0.05	3.411 (7)
O(1)	8*c*	0.25	0.25	0.25	0.22(1)	5.0(2)
O(2)	32*f*	0.314(1)	0.314(1)	0.314(1)	0.109(3)	5.0(2)
O(3)	48*i*	0.5	0.25	0.25	0.109(3)	5.0(2)

Table S11 Crystallographic data and refinement parameters for the $Bi_{1.7}Dy_{0.2}Er_{0.075}W_{0.0125}O_3$ composition at ambient temperatures from PDF fitting. Fitting range 4.5-50 Å.

Composition	$Bi_{1.7}Dy_{0.2}Er_{0.075}W_{0.0125}O_3$
R_w (%)	3.8
Formular weight	906.210
Space group	*Fm*-3*m*
a /Å	5.55065(3)
Cell volume /$Å^3$	168.878
Z	2
Crystal density /$g.cm^{-3}$	8.7992(1)

Table S12 Refined structural parameters for the $Bi_{1.7}Dy_{0.2}Er_{0.075}W_{0.0125}O_3$ composition at ambient temperatures.

Atom	Site	*x*	*y*	*z*	*Occ*	B_{iso} (Å^2)
Bi	4*a*	0	0	0	0.8556	3.390(6)
Dy	4*a*	0	0	0	0.1007	3.390(6)
Er	4*a*	0	0	0	0.0378	3.390(6)
W	4*a*	0	0	0	0.0062	3.390(6)
O(1)	32*f*	0.300(1)	0.300(1)	0.300(1)	0.087(6)	5.0(1)
O(2)	48i	0.5	0.195(2)	0.195(2)	0.018(1)	5.0(1)

Table S13 Crystallographic data and refinement parameters for the $Bi_{1.7}Dy_{0.2}Er_{0.075}Nb_{0.025}O_{3.025}$ composition at ambient temperatures from PDF fitting. Fitting range 4.5-50 Å

Composition	$Bi_{1.7}Dy_{0.2}Er_{0.075}Nb_{0.025}O_{3.025}$
R_w (%)	3.9
Formular weight	902.064
Space group	*Fm*-3*m*
a /Å	5.54158 (3)
Cell volume /Å^3	168.878
Z	2
Crystal density /g.cm^{-3}	8.5862(1)

Table S14 Refined structural parameters for the $Bi_{1.7}Dy_{0.2}Er_{0.075}Nb_{0.025}O_{3.025}$ composition at ambient temperatures from PDF fitting.

Atom	Site	*x*	*y*	*z*	*Occ*	B_{iso} (Å^2)
Bi	4*a*	0	0	0	0.85	3.320 (6)
Dy	4*a*	0	0	0	0.10	3.320 (6)
Er	4*a*	0	0	0	0.0375	3.320 (6)
Nb	4*a*	0	0	0	0.0125	3.320 (6)
O(1)	32*f*	0.300(1)	0.300(1)	0.300(1)	0.136(5)	5.75(64)
O(2)	48*i*	0.5	0.202(8)	0.202(8)	0.006(1)	5.75(64)

Table S15 Crystallographic data and refinement parameters for the $Bi_{1.7}Dy_{0.2}Nb_{0.075}W_{0.025}O_{3.075}$ composition at ambient temperatures from PDF fitting. Fitting range 4.5-50 Å.

Composition	$Bi_{1.7}Dy_{0.2}Nb_{0.075}W_{0.025}O_{3.075}$
R_w (%)	3.9
Formular weight	898.258
Space group	*Fm*-3*m*
a /Å	5.54375(3)
Cell volume /Å^3	169.934
Z	2
Crystal density /g.cm^{-3}	8.7546(1)

Table S16 Refined structural parameters for the $Bi_{1.7}Dy_{0.2}Nb_{0.075}W_{0.025}O_{3.075}$ composition at ambient temperatures from PDF fitting.

Atom	Site	x	y	z	*Occ*	B_{iso} (Å^2)
Bi	4a	0	0	0	0.85	3.167(6)
Dy	4a	0	0	0	0.10	3.167(6)
Nb	4a	0	0	0	0.0375	3.167(6)
W	4a	0	0	0	0.0125	3.167(6)
O(1)	32f	0.304(1)	0.304(1)	0.304(1)	0.136(3)	5.0(1)
O(2)	48i	0.5	0.195(4)	0.195(4)	0.010(1)	5.0(1)

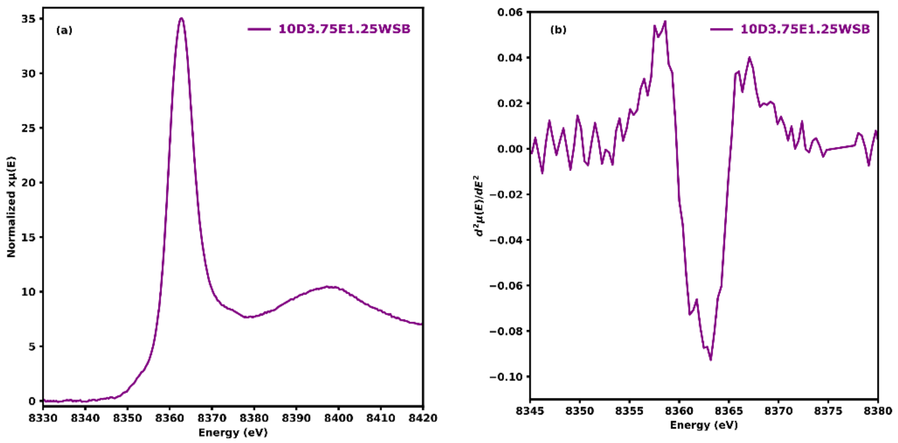

Figure S7 **(a)** Normalized Er L_3-edge XANES and **(b)** second derivative of the normalized XANES spectrum of Er^{3+} in the $Bi_{1.7}Dy_{0.2}Er_{0.075}W_{0.0125}O_3$ composition at ambient temperature.

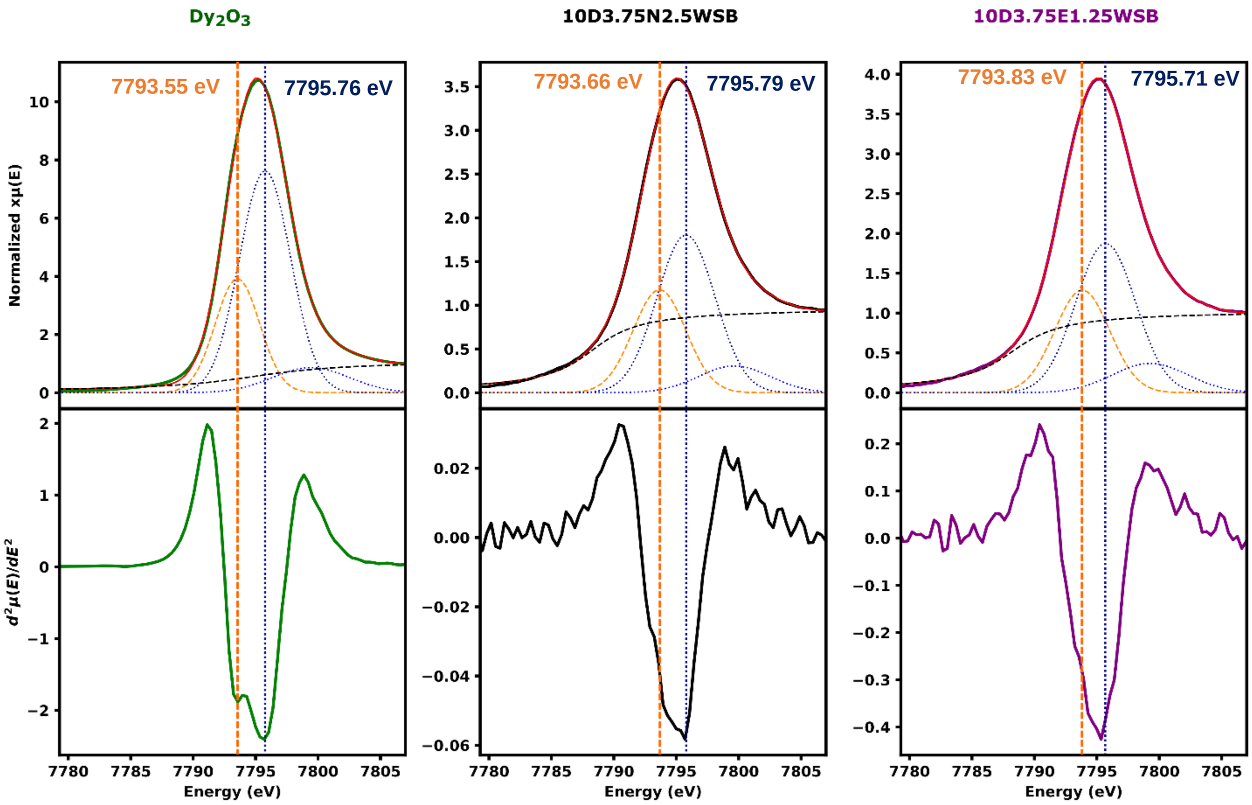

Figure S8 Normalized and fitted Dy L_3-edge white line sharing the same x-axis with the second derivative of the Dy L_3-edge normalized XANES spectrum of Dy_2O_3, $Bi_{1.7}Dy_{0.2}Nb_{0.075}W_{0.025}O_{3.075}$, $Bi_{1.7}Dy_{0.2}Er_{0.075}W_{0.0125}O_3$, compositions at ambient temperature.

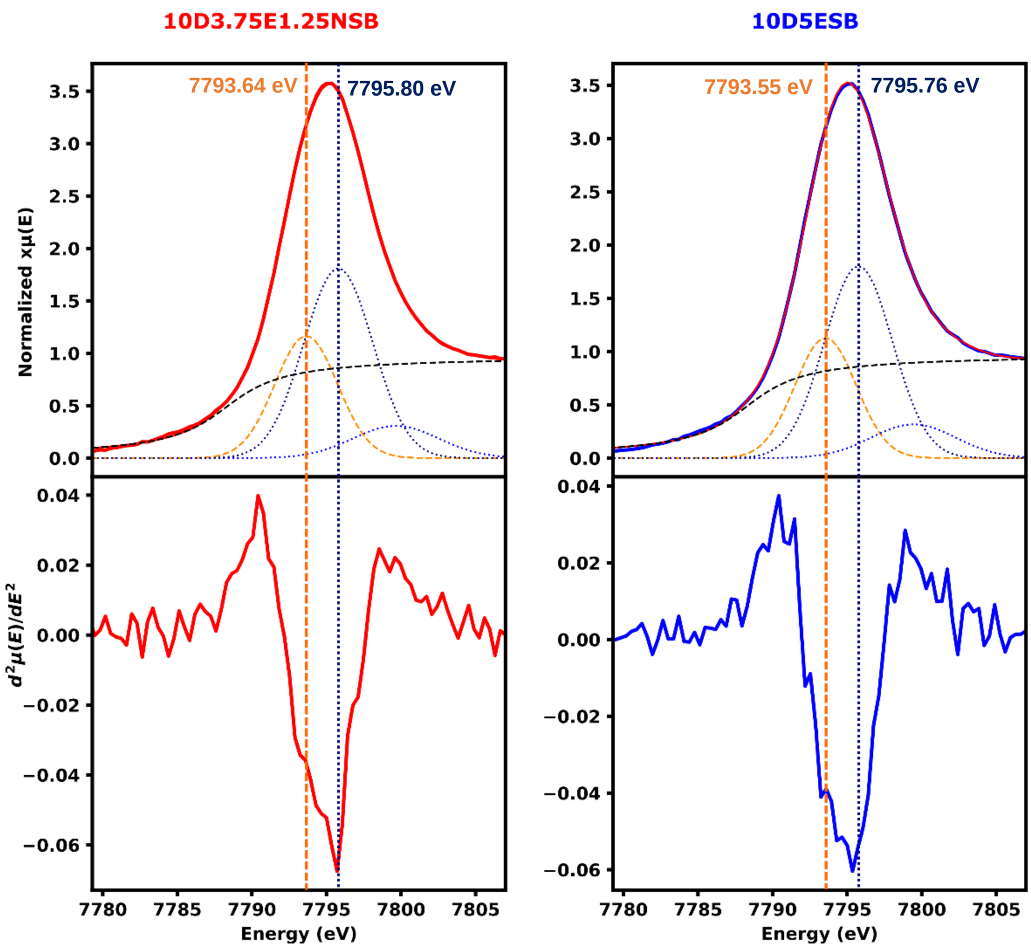

Figure S9 Normalized and fitted Dy L_3-edge white line sharing the same x-axis with the second derivative of the Dy L_3-edge normalized XANES spectrum of $Bi_{1.7}Dy_{0.2}Er_{0.075}Nb_{0.025}O_{3.025}$, and $Bi_{1.7}Dy_{0.2}Er_{0.1}O_3$ compositions at ambient temperature.

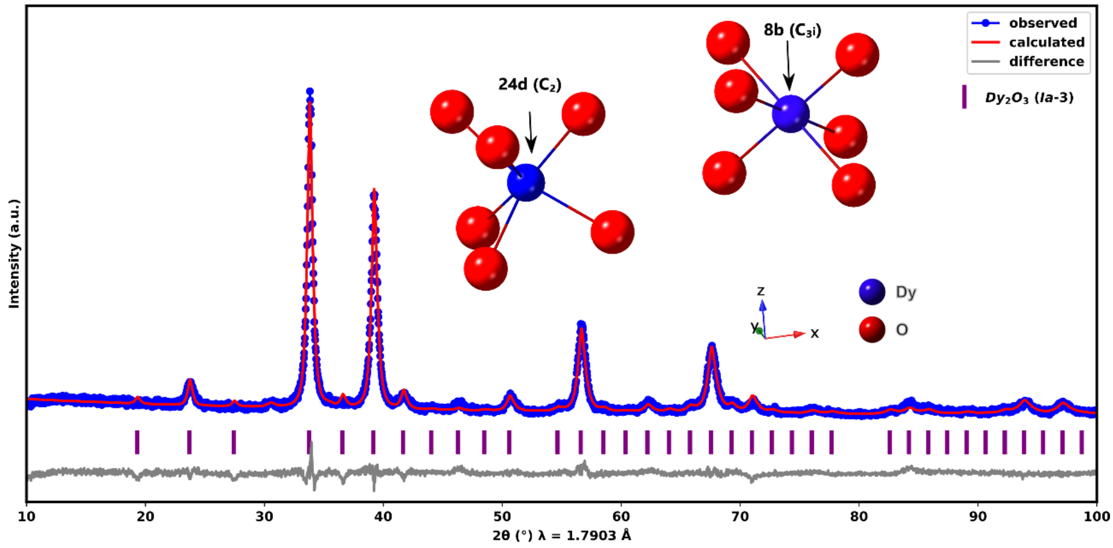

Figure S10 Rietveld refinement of the Dy_2O_3 sample at ambient temperatures. Purple hkl ticks are for the Dy_2O_3 phase (I*a*-3) obtained from the Antic model (Antic et al., 1993). The inset show the two different sites of the Dy^{3+} cations found in the I*a*-3 model.

Table S17 Crystallographic data of Dy_2O_3 at ambient temperature. R_{wp} = 9.29% and goodness of fit (GOF) =1.44.

Atom	Site	*x*	*y*	*z*	*Occ*	B_{iso} (Å^2)
Dy(1)	8*b*	0.25	0.25	0.25	1.00	1.6 (2)
Dy(2)	24*d*	-0.0264(2)	0	0.25	1.00	1.6 (2)
O(1)	48*e*	0.484(2)	0.181(1)	0.254(1)	1.00	5.0(7)

Table S18 Refined structural parameters for the Dy_2O_3 at ambient temperatures.

Phases	Bixbyite (I*a*-3)
Weight %	100
Space group	*Ia*-3
a /Å	10.6743(3)
Cell volume /Å^3	31216.2(1)
Crystal density /g.cm^{-3}	8.1481(7)

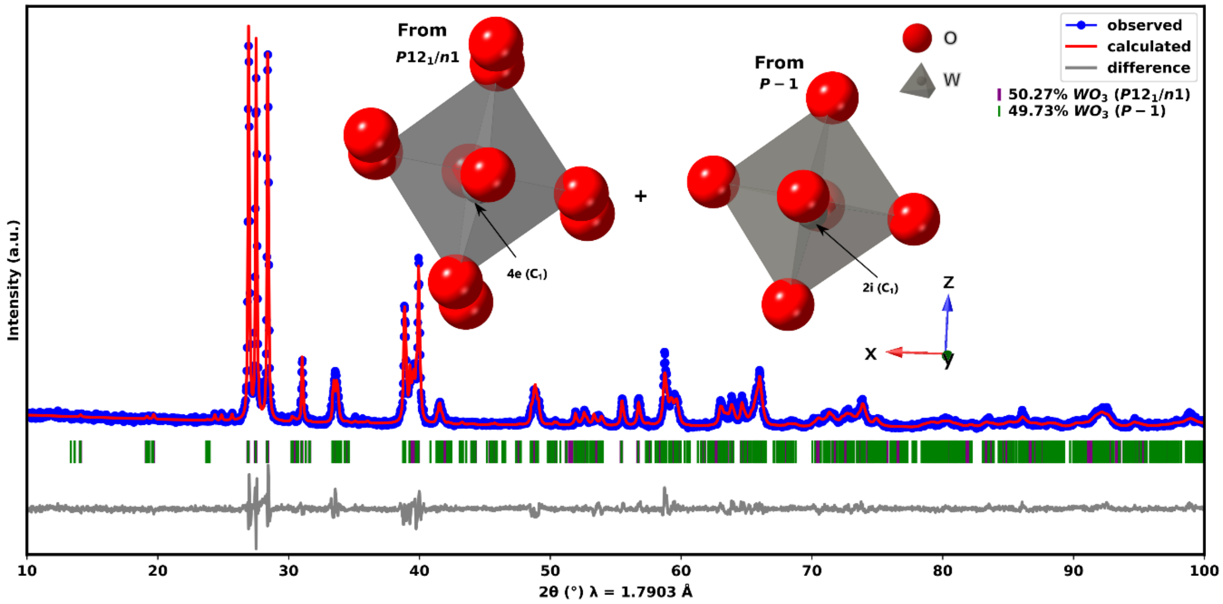

Figure S11 Rietveld refinement of the WO_3 sample at ambient temperatures. Purple and green hkl ticks are for the WO_3 ($P12_1/n1$) and WO_3 phases (P-1) obtained from the Vogt (Vogt et al., 1999) and (Diehl, R. & Brandt, G, 1978) models respectively. The inset shows the distorted octahedra of WO_3 found in some of the sites in these models.

Table S19 Crystallographic data of WO_3 composition at ambient temperature. R_{wp} =10.46% and GOF = 1.54

Phases	Monoclinic ($P12_1/n1$)	Triclinic(P-1)
Weight %	50(4)	50(4)
Space group	$P12_1/n1$	P-1
a /Å	7.301(1)	7.305(1)
b /Å	7.5347(9)	7.5317(8)
c /Å	7.692(1)	7.692(1)
α	90	89.770(8)
β	90.13(1)	90.677(6)
γ	90	89.98(1)
Cell volume /$Å^3$	423.1(1)	423.15(9)
Crystal density /$g.cm^{-3}$	7.278(1)	7.278(1)

Table S20 Refined structural parameters for the WO_3 ($P12_1/n1$) at ambient temperatures.

Atom	Site	*x*	*y*	*z*	*Occ*	B_{iso} (Å^2)
W(1)	4*e*	0.255(5)	0.009(4)	0.267(2)	1.00	4.9(3)
W(2)	4*e*	0.259(5)	0.010(5)	0.749(3)	1.00	4.9(3)
O(1)	4*e*	-0.05(2)	0.07(2)	0.25(7)	1.00	0.003
O(2)	4*e*	0.01(4)	0.51(3)	0.25 (7)	1.00	0.003
O(3)	4*e*	0.50(3)	-0.01(3)	0.25(4)	1.00	0.003
O(4)	4*e*	0.25(5)	0.26(4)	0.75(3)	1.00	0.003
O(5)	4*e*	0.25(4)	-0.04(4)	0.05(1)	1.00	0.003
O(6)	4*e*	0.26(3)	0.54(4)	-0.03(1)	1.00	0.003

Table S21 Refined structural parameters for the WO_3 (P-1) at ambient temperatures.

Atom	Site	*x*	*y*	*z*	*Occ*	B_{iso} (Å^2)
W(1)	2*i*	0.253(6)	0.038(5)	0.293(6)	1.00	0.001
W(2)	2*i*	0.255(6)	0.538(5)	0.212(6)	1.00	0.001
W(3)	2*i*	0.254(6)	0.039(5)	0.795(7)	1.00	0.001
W(4)	2*i*	0.253 (6)	0.535(5)	0.710(6)	1.00	0.001
O(1)	2*i*	0.23(4)	0.01(5)	-0.01(5)	1.00	0.001
O(2)	2*i*	0.58(2)	0.40(2)	0.20(2)	1.00	0.001
O(3)	2*i*	-0.15(2)	0.35(2)	0.42(2)	1.00	0.001
O(4)	2*i*	0.54(2)	0.94(3)	0.25(2)	1.00	0.001
O(5)	2*i*	0.24(4)	0.25(2)	0.31(3)	1.00	0.001
O(6)	2*i*	0.20(4)	0.75(3)	0.22(3)	1.00	0.001
O(7)	2*i*	0.16(3)	0.31(3)	0.74(3)	1.00	0.001
O(8)	2*i*	0.27(4)	0.74(3)	0.76(4)	1.00	0.001
O(9)	2*i*	0.23(4)	0.23(3)	1.18(3)	1.00	0.001
O(10)	2*i*	0.56(2)	0.55(2)	0.34(2)	1.00	0.001
O(11)	2*i*	0.24(3)	0.51(3)	1.00(3)	1.00	0.001
O(12)	2*i*	0.23 (4)	1.02(5)	0.51(5)	1.00	0.001

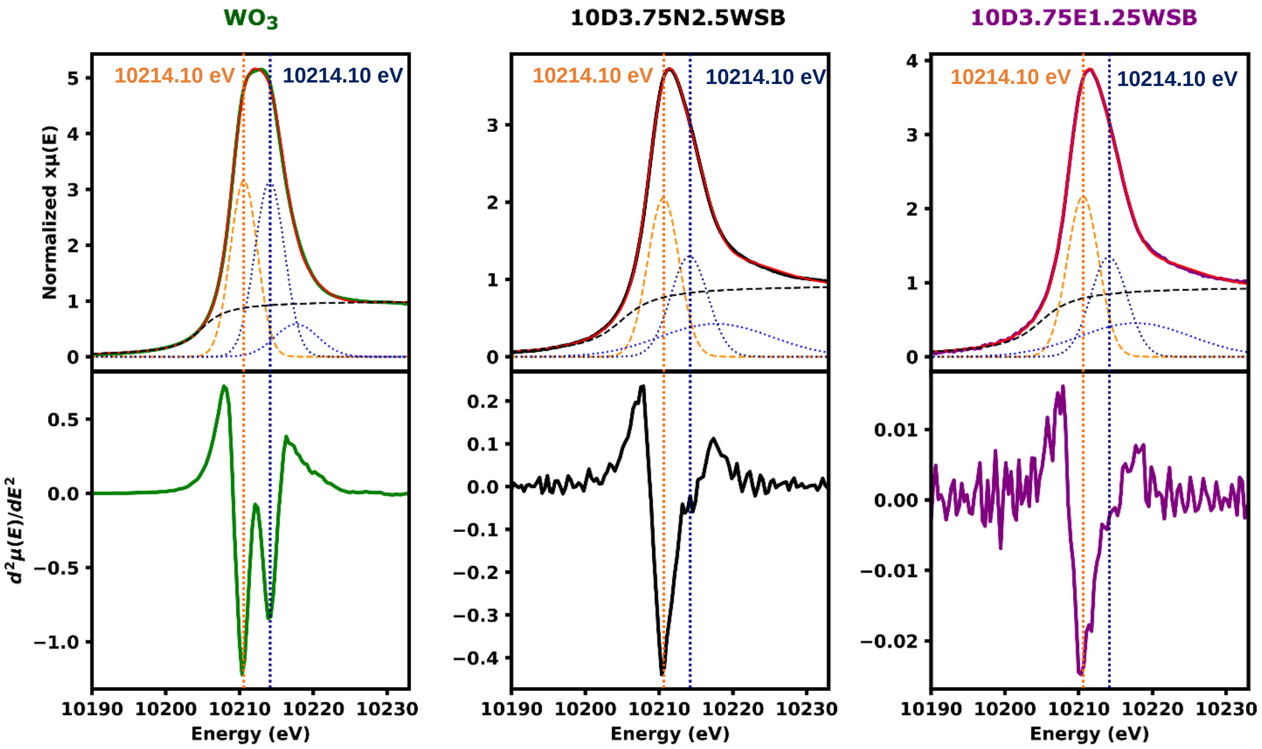

Figure S12 Normalized and fitted W L_3-edge white line sharing the same x-axis with the second derivative of the W L_3-edge normalized XANES spectrum of WO_3, $Bi_{1.7}Dy_{0.2}Nb_{0.075}W_{0.025}O_{3.075}$, $Bi_{1.7}Dy_{0.2}Er_{0.075}W_{0.0125}O_3$, compositions at ambient temperature.

References

Antic, B., Önnerud, P., Rodic, D., & Tellgren, R. (1993) **8**, 216–220.

Hull, S., Norberg, S. T., Tucker, M. G., Eriksson, S. G., Mohn, C. E., & Stølen, S. (2009). *Dalton Transactions* **40**, 8737–8745.

Leszczynska, M., Liu, X., Wrobel, W., Malys, M., Krynski, M., Norberg, S. T., Hull, S., Krok, F., & Abrahams, I. (2013). *Chemistry of Materials* **25**, 326–336.

Vogt, T., Woodward, P. M., & Hunter, B. A. (1999. *Journal of Solid State Chemistry* **144**, 209-215.

Diehl, R. & Brandt, G (1978). *Acta Cryst.B34, 1105-1111*
